# FastSpel: A Method for Fast Spectral Library Generation

**DOI:** 10.1021/acs.jproteome.5c00279

**Published:** 2025-08-27

**Authors:** Mehdi B. Hamaneh, Yi-Kuo Yu

**Affiliations:** Division of Intramural Research, National Library of Medicine, 10952 National Institutes of Health, Bethesda, Maryland 20894, United States

**Keywords:** mass spectrometry, spectrum prediction, rescoring

## Abstract

Recently, several methods have been proposed for predicting peptide MS/MS fragment intensity profiles. These predicted profiles may be used for generating spectral libraries for data-independent acquisition analysis, or for improving peptide identification by rescoring the peptide-spectra matches identified by search engines such as MaxQuant. Although some of the proposed intensity prediction methods generate high quality spectral libraries and significantly improve peptide identification, they are computationally expensive and their parameters are difficult to interpret. In this paper, we introduce FastSpel (fast spectral library), a fast and interpretable fragment intensity prediction method for tryptic peptides. Testing FastSpel on 23 independent data sets, we show that its performance, in terms of improving peptide identification via rescoring and spectral library generation, is comparable with those of the existing state-of-the-art methods, while being over 2 orders of magnitude less computationally expensive than these methods. Moreover, analysis of parameters of the model corroborates known fragmentation rules, such as the “proline effect”, and suggests novel patterns. In addition to FastSpel, we propose a simple scoring function that achieves rescoring/identification performance close to that of Percolator, a widely used program for this purpose, without requiring model training as Percolator does.

## Graphical abstract

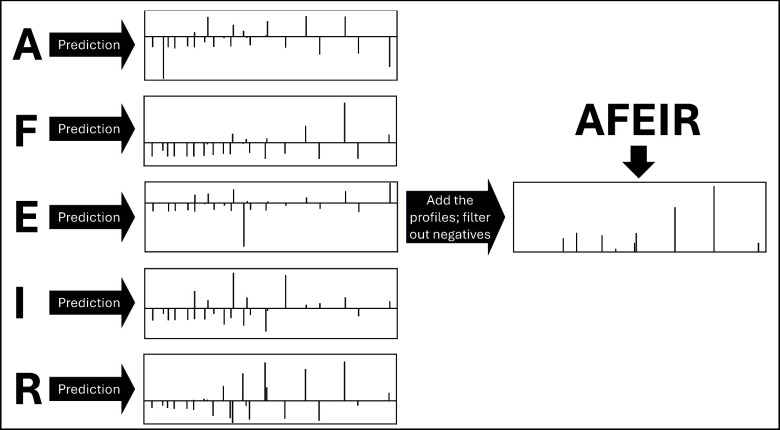

## Introduction

In mass spectrometry-based proteomics, in silico-predicted peptide MS/MS fragment intensities may improve peptide identification via rescoring and can be used in place of a spectral library for application, for instance, in data-independent acquisition (DIA) analysis. Given the importance of these applications, many fragment intensity prediction methods have been developed, some of which date back more than two decades.^1,2^ Especially, in recent years several machine learning-based predictors have been proposed including MS2PIP,^3^ AlphaPeptDeep^4^ (PeptDeep for short), Prosit Transformer,^5^ DeepMass:Prism,^6^ pDeep3,^7^ Prosit,^8^ DeepDIA,^9^ PredFull,^10^ and the method proposed by Guan et al.^11^

Although some of the proposed deep learning-based methods achieve high accuracy,^12^ they are computationally expensive and their parameters are notoriously hard to interpret. In this paper, as a proof of concept, we introduce FastSpel (fast spectral library), a simple, fast, and interpretable method trained for predicting fragment intensities for tryptic peptides and higher-energy collisional dissociation (HCD) fragmentation type. Using 22 testing data sets, we demonstrate that, despite being slightly less accurate, in terms of the number of peptides identified via rescoring, FastSpel performs comparably with PeptDeep, Prosit, and MS2PIP, while being over 2 orders of magnitude less computationally expensive than these methods (when run on the same system). Additionally, performing DIA analysis (using DIA-NN^13^) on another data set, we show that the FastSpel-generated spectral library results in identification of essentially the same peptides as the ones identified using the spectral libraries created by PeptDeep, Prosit, or MS2PIP. We chose these three methods for comparison for a couple of reasons. First, PeptDeep and especially Prosit are among the most widely used predictors.^14^ They have also been reported to have the highest accuracy measured by both similarity between the corresponding predicted/observed profiles,^12^ and the number of identified peptides via rescoring.^4,8^ On the other hand, MS2PIP, a less sophisticated method, has been shown to perform comparably with Prosit in terms of peptide identification using Percolator.^8^ Second, these three methods can be run through the same rescoring package Oktoberfest,^15^ which provides a consistent way of assessing the methods.

Another advantage of FastSpel is the interpretability of its parameters. FastSpel uses a multistep linear regression to relate the matrix representing the sequences of peptides of given length and charge, to the matrix containing the predicted fragment intensities. This simple linear relation allows us to easily interpret the model parameters and relate them to known rules of fragmentation chemistry (e.g., the “proline effect”), or suggest new fragmentation patterns.

In addition to FastSpel, we propose a new scoring function that is the product of two features calculated based on the predicted intensities: the number *n*_op_ of peaks that are both observed and predicted, and normalized (spectral) angle, defined as α = 1 – (2θ/π) with θ being the angle between the corresponding predicted and observed intensity vectors.^5,12,16^ Our results show that (1) the proposed score performs nearly as well as Percolator in peptide identification without the need for training a model using a portion of the data, and (2) FastSpel performs comparably with or better than the three alternative methods, when the proposed score alone is used for rescoring and identifying peptides. Put together, these two findings imply that the good performance of FastSpel + Percolator is *not* because of Percolator, it is rather because of the good quality of the intensity profiles predicted by FastSpel.

Using a single (AMD EPYC 3.8 GHZ) CPU, FastSpel is able to predict intensities for one million precursors in less than 5 s. Hence, the method can be of practical importance whenever computational cost is a concern. More importantly, because of its high prediction speed, FastSpel in conjunction with the proposed scoring function may be used for on-the-fly prediction and scoring, which may be beneficial for database search or de novo peptide sequencing. However, investigating the potential benefits of using FastSpel for these purposes requires extensive testing and is beyond the scope of this paper.

## Methods

### Data

#### Training Data

We used the ProteomeTools^17^ data for training FastSpel. The tryptic, HCD data were downloaded from https://figshare.com/projects/Prosit/35582. The data are already divided into training and testing sets, which we henceforth refer to as PT-train and PT-test data, respectively. The PT-train data were used for training, but the PT-test data were used only to check for overfitting. For actually testing FastSpel, 23 independent data sets, comprising 22 data-dependent acquisition (DDA) and 1 DIA data sets, were used as described below. The PT-train data contain peptides of lengths between 7 and 30, charges between 1 and 6, and intensities obtained using 6 collision energies that are 20, 23, 25, 28, 30, and 35. Our model was therefore trained for these ranges of values. Of note, the ProteomeTools data are already preprocessed and ready to use, that is the peptide spectrum matches (PSMs) are already identified and *b*/*y* fragment intensities are extracted. Thus, no preprocessing was performed on the data.

#### DDA Testing Data (Rescoring)

For testing FastSpel, in terms of improving peptide identification via rescoring, and comparing its performance with those of the other methods, 22 DDA data sets were downloaded from the PRIDE repository.^18^ These constitute a subset of the ones used by the authors to compare six intensity prediction methods in a recent publication, which includes a more detailed description of the selection criteria.^12^ Specifically, only HCD data sets were included and CID (collision-induced dissociation) data were excluded.

The 22 PRIDE data sets used for testing FastSpel rescoring performance are listed in Table 1. These include data obtained from different species and using two different family of MS instruments (Q Exactive and Fusion). In the rest of the paper, we refer to the Q Exactive instruments (including Q Exactive HF and Q Exactive Plus) as QE. Also, both Fusion and Fusion Lumos instruments are referred to as Fusion.

**Table 1:** Datasets Used for Testing FastSpel^a^

PRIDE ID	organism	CE	instrument
PXD012025	Drosophila melanogaster (fruit fly)	27	QE
PXD013270	Rattus norvegicus (rat)	27	QE
PXD014374	Bos taurus (bovine)	28	QE
PXD015442	Rattus norvegicus (rat)	30	Fusion
PXD016793	Rattus norvegicus (rat)	27	QE
PXD019296	Rattus norvegicus (rat)	27	QE
PXD020557	Homo sapiens (human)	27	QE
PXD020756	Bos taurus (bovine)	30	QE
PXD021588	Homo sapiens (human)	26	QE
PXD026323	Saccharomyces cerevisiae (baker’s yeast)	28	QE
PXD031222	Proteus mirabilis HI4320 (bacterium), Candida albicans (yeast)	27/30	QE
PXD032834	Plasmodium falciparum (isolate 3d7) (malaria parasite)	25/28	QE
PXD033348	Saccharomyces cerevisiae (baker’s yeast)	27	QE
PXD034156	Mus musculus (mouse)	27	QE
PXD035632	Synechocystis sp. PCC 6803 (bacterium)	26/27	QE
PXD035677	Arabidopsis thaliana (mouse-ear cress)	30	Fusion
PXD036475	Escherichia coli (bacterium)	35	Fusion
PXD036796	Staphylococcus aureus (bacterium)	28	QE
PXD040423	Mus musculus (mouse)	30	Fusion
PXD040724	Caenorhabditis elegans (nematode)	25	QE
PXD041442	Mus musculus (mouse)	27	QE
PXD042301	Mus musculus (mouse)	27	QE

a CE: collision energy.

#### DIA Testing Data

A three species (human, yeast, and *E. coli*) benchmark data set (PXD028735)^19^ with known relative protein abundances, was used for evaluating FastSpel in regards to spectral library generation for DIA analysis. We included only raw files containing the mixture of the three species and obtained using all-ion fragmentation and QE, as FastSpel is not currently trained for timsTOF instruments (26 raw files). These raw files are grouped under “condition A” (CA), “condition B” (CB), and “QC”, each group having different quantities of the three species.

#### Identifying PSMs for Rescoring (DDA Data)

MaxQuant^20^ version 2.1.3.0 was employed to find PSMs for each of the 22 DDA tryptic PRIDE projects (Table 1) under two scenarios: (1) both protein and peptide false discovery rates (FDRs) were set to 1% to identify true PSMs, and (2) the FDRs were chosen to be 100% to generate both target and decoy PSMs. The rest of the parameters were set to the default values. For each PRIDE project, we used the provided fasta file(s). If no fasta file was provided, the fasta file from a representative proteome, downloaded from https://www.uniprot.org/proteomes,^21^ was used.

The target PSMs identified in the runs with FDR = 1% were used to evaluate FastSpel in terms of the similarity between the peptide intensity profiles predicted by FastSpel and the matching spectra. These true PSMs were also used for calibrating FastSpel to be used for rescoring (“Collision Energy Calibration” subsection). The target and decoy PSMs found in the MaxQuant runs with FDR = 100% were rescored using FastSpel and Oktoberfest to improve peptide identification (see the “Rescoring and Peptide Identification” subsection). During the rescoring process, Oktoberfest extracts and saves the experimental (observed) intensity profiles from the raw files for all PSMs (targets and decoys obtained in MaxQuant runs with FDR = 100%). The experimental intensity profiles reported by Oktoberfest were used in all analyses performed in this study.

### Description of FastSpel Prediction Method

#### Prediction

For *N*_*lze*_ peptides of charge *z* and length *l*, we use a multistep linear regression to find an equation that relates the matrix containing the predicted intensity profiles (for collision energy *e*) to the matrix representing the corresponding peptides’ sequences. Mathematically, this can be written as

1 $$I_{lze}=S_{lze}X_{lze}$$

where *I*_*lze*_, and *S*_*lze*_ are respectively the matrices representing the predicted intensities and the peptides’ sequences, and *X*_*lze*_ is an unknown matrix to be found using the multistep linear regression and the training data (see the “Training” subsection). Since the intensities are positive, any negative value in *I*_*lze*_ is replaced by zero to get the final predicted intensity matrix.

The procedure for constructing *S*_*lze*_ from sequences has been explained in detail in Text S1 and is visualized in Figure S1. Briefly, each row of *S*_*lze*_ is a 1 × *M*_*l*_ vector (containing zeros and ones) that encodes a peptide sequence, making *S*_*lze*_ an *N*_*lze*_ × *M*_*l*_ matrix. Here *M*_*l*_ = *ml*, with *m* being the number of amino acids the peptides are composed of. In this study, we consider only peptides that consist of a subset of the 20 prevalent amino acids plus oxidized methionine (overall *m* = 21 amino acids). Corresponding to each row of *S*_*lze*_, there is a row in *I*_*lze*_ that contains the predicted fragment intensities for the peptide encoded in that row of *S*_*lze*_. The fragment intensities are calculated up to maximum fragment charge *z*_f_max__ = min­(3,*z*), making *I*_*lze*_ an *N*_*lze*_ × *K*_*lz*_ matrix, where *K*_*lz*_ = 2­(*l* – 1)*z*_f_max__ is the number of theoretically possible peaks. More details regarding building *I*_*lze*_ are provided in Text S1.

Of note, each column of *X*_*lze*_, which is an *M*_*l*_ × *K*_*lz*_ matrix, corresponds to one of the *b* or *y* fragments. On the other hand, each row of *X*_*lze*_ is associated with an amino acid/position combination. As examples, assuming the amino acids are ordered as shown in Figure S1, the first and 46th rows of *X*_*lze*_ correspond, respectively, to alanine in the first position (N terminus) and glutamic acid in the third position in the sequence. Thus, in our model the intensity profile of a given peptide with *lze* values is the sum of the rows of *X*_*lze*_ that correspond to the amino acid/position combinations present in the peptide. (Of course, after adding these rows, one needs to replace any resulting negative value by zero). In other words, each element of *X*_*lze*_ can be interpreted as the contribution of a certain amino acid/position combination to the intensity of a certain ion type.

#### Training

Given some training data represented by the sequence matrix *S*_*lze*_^*t*^ and the intensity matrix *I*_*lze*_^*t*^, the matrix *X*_*lze*_ is found by a multistep linear regression method (superscript *t* indicates the matrices are generated using the training data). The details of this approach, and the rationale behind it, are discussed in Texts S2–S4. The workflow is also depicted in Figure S2. Briefly, in step *n* all zero values in the experimental intensity matrix *I*_*lze*_^*t*^ are replaced by −(*n* – 1)­0.05, and eq 1 is solved (in the least-squares sense) for *X*_*lze*_. The multistep process is terminated when there is no improvement in the prediction accuracy, measured by the median cosine of the angles between the predicted and corresponding experimental intensity vectors.

Note that eq 1 is solved only if it is overdetermined, that is if *N*_*lze*_ is larger than the number of linearly independent rows of *S*_*lze*_^*t*^. This criterion is satisfied if *N*_*lze*_/(*ml*) ≥ 1 (see Text S3). However, since there may be many redundant peptides in the training data, we require $r_{lze}={Ñ}_{lze}/\left(ml\right)\geq 1$ , where ${Ñ}_{lze}$ is the number of nonredundant peptides with *l*/*z*/*e* values. (If *r*_*lze*_ < 1 FastSpel is not trained for *l*/*z*/*e*.) This requirement means that, for FastSpel to be trained, we need ${Ñ}_{lze}\geq ml$ . For example, for peptides of length 20 we need at the absolute minimum 420 (*ml* = 21*20) nonredundant peptides with *lze* values to train the model. If *r*_*lze*_ is only slightly larger than 1, overfitting may happen and more nonredundant peptides may be needed for better accuracy. We address this issue in Results and Discussion. It is worth mentioning that oxidized and unmodified methionines are treated as two different amino acids in our model, and so peptides with an oxidized methionine are considered nonredundant to their nonoxidized counterparts.

#### Collision Energy Calibration

Since the training data may not cover many collision energy values, if the test data set has been obtained using a collision energy for which the model has not been trained, we use the *X*_*lze*_ with closest *e* to that of the test data set. Alternatively, one can perform collision energy calibration if some known true PSMs are available. Considering a range of collision energies, the calibration process predicts fragment intensities for peptides with known true spectra matches. These predictions are then compared with the experimental intensities of the matching spectra and the optimal collision energy, that is the one that maximizes the similarity between the predicted and experimental intensities, is identified. This optimal value is then used for rescoring. Before rescoring peptides, both Prosit and PeptDeep (as implemented in Oktoberfest) use the 1000 top-scoring PSMs (scored by MaxQuant) to perform collision energy calibration.

Given MaxQuant search results, FastSpel can be calibrated in the same manner, except that the collision energies considered are limited to the values the method has been trained for (20, 23, 25, 28, 30, 35), and that FastSpel is calibrated separately for each *l* and *z* combination using all PSMs at the FDR = 1% level in all raw files (FastSpel is not calibrated for each raw file separately). Of note, MS2PIP does not accept collision energy as an input, and thus is not calibrated.

### The Proposed Scoring Function

Rescoring pipelines like Oktoberfest compute a bunch of features for each peptide that are then used as input to Percolator for rescoring the peptides. These features include some general properties like peptide length/charge etc. (general features), intensity-related features that are the ones calculated using predicted intensities, and features computed based on predicted retention times, which we refer to as retention time-related (RT-related) features. The intensity-related features can be divided into two categories: (1) features computed using the magnitude of the intensities, and (2) features calculated based only on the presence (being nonzero) or absence (being zero) of the peaks. In Oktoberfest, the first category includes only two features, namely normalized (spectral) angle α and correlation *R* between the predicted and observed intensities. The rest (vast majority) of the features are in the second category, which includes the number *n*_o_ of the observed peaks, the number *n*_p_ of the (nonzero) predicted peaks, the number *n*_op_ of peaks that are both observed and predicted, the overlap *f* = *n*_op_/*n*_p_, and many more. Because there are only two categories of intensity-related features, we hypothesized that a simple score/feature that is the product of one feature from category (1) and one feature from category (2) could perform well, if used as a single score/feature for rescoring. Since α has been argued to be a better similarity measure than *R*,^8^ we chose α from category (1). On the other hand, the overlap *f* = *n*_op_/*n*_p_ has been reported to be a powerful discriminant between targets and decoys.^8^ Therefore, in category (2), we narrowed our focus on features that are calculated based on *n*_op_. In addition to overlap *f* = *n*_op_/*n*_p_, there is only one feature that satisfies this criterion, and that is *n*_op_ itself. Thus, we considered these two features from category (2), resulting in two candidates for the new scoring function: α*n*_op_ and α*f*.

### Running Prosit, PeptDeep, and MS2PIP

We employed Oktoberfest version 0.7.0 to run Prosit, PeptDeep, and MS2PIP. The latest respective prediction models were used for prediction of intensities (“Prosit-2020-intensity-HCD”, “AlphaPept-ms2-generic”, “ms2pip-2021-HCD”) and retention times (“Prosit-2019-irt”, “AlphaPept-rt-generic”, “Deeplc-hela-hf”^22^). For collision energy calibration a range of 20 ≤ *e* ≤ 40 was used. Of note, MS2PIP does not accept collision energy as an input, and so collision energy calibration procedure, which is performed automatically by Oktoberfest, does not have any effect on the intensities predicted MS2PIP. In each Oktoberfest run, the number of CPUs (the “numThreads” parameter) was set to 8. All other parameters were set to the default values.

### Rescoring and Peptide Identification

#### Rescoring Using a Single Score/Feature

For rescoring and peptide identification using a single feature/score, such as normalized angle α or overlap *f*, the PSMs were first sorted, in descending order, based on the feature/score. Redundant peptides were then removed and only the highest ranking peptide was kept. This sorted list of peptides and the corresponding list of target/decoy labels were then used for FDR estimation. For consistency, we employed the “calculate-fdrs” function in Oktoberfest to compute the FDRs.

#### Rescoring with Percolator

It has been reported that Percolator may fail to correctly control the FDR.^23^ To avoid this issue, the Percolator-RESET algorithm has been recently proposed.^24^ Thus, instead of using the results of Percolator called automatically by Oktoberfest, we ran the Percolator-RESET algorithm implemented in Percolator version 3.07.01. (For brevity, in the rest of the paper we use Percolator to refer to Percolator-RESET.) Since FastSpel does not currently predict retention times, Percolator was run both including and excluding RT-related features. We denote the former by “Percolator (IGRTC)”, and the latter by “Percolator (IGC)”. Here, G, I, and RT stand for General, intensity-related, and RT-related, respectively. The “C” in IGRTC and IGC indicates that the intensities are calculate using the collision energy calibrated methods (not applicable to MS2PIP).

To study the effect of collision energy calibration on the results, we also ran Percolator using the features computed based on the predictions of the uncalibrated methods. Since running the rescoring pipeline without collision energy calibration is not an available option in Oktoberfest, we ran Oktoberfest in the “spectral library generation” mode to get the predicted intensities, which were then fed to the relevant functions of Oktoberfest to compute the intensity-related features. The intensity-related and general features were then used to run Percolator. We henceforth refer to this run as “Percolator (IG)”. Detailed information about how Percolator was run are given in Text S5.

### DIA Analysis

To evaluate the spectral libraries generated by FastSpel we employed DIA-NN^13^ to identify/quantify peptides/proteins using data from PXD028735. The UniProt^21^ fasta files of human, yeast, and *E. coli* were concatenated into a single fasta file to be used for digestion by Oktoberfest and DIA-NN. Oktoberfest was then run, in the “spectral library generation” mode to generate libraries using Prosit, PeptDeep, and MS2PIP. Oktoberfest produces an input file containing precursors for prediction. For consistency, we used this input file for library generation by FastSpel. Also, retention times predicted by Prosit were used for all four methods to make sure any observed differences in the identification/quantification results are due only to differences in the generated libraries. The library generated by each method (containing 27285296 spectra) was then used as input to run DIA-NN. When running DIA-NN, the *q*-value cutoff for identification, the maximum number of missed cleavages, and the fragment filtering mass accuracy, were respectively set to 0.01, 2, and 20 ppm. The “reanalyse” option was used to enable “match between runs”. To calculate the log fold changes, the intensities reported by DIA-NN were first log-transformed and then averaged over the samples belonging to each condition. Given a pair of conditions, for each species, the fold changes were then calculated by subtracting the corresponding average log-transformed intensities.

## Results and Discussion

This section is organized as follows. First, we provide details regarding the training process and interpret the model parameters (elements of the *X*_*lze*_ matrices). Then, FastSpel is evaluated and compared with Prosit, PeptDeep, and MS2PIP in terms of similarity between the corresponding predicted/observed intensities, rescoring, DIA search, and prediction speed. Finally, limitations of FastSpel are discussed. Of note, since many mathematical symbols introduced in Methods are used in this section, as a quick reference, a list of these symbols and their descriptions are given in Text S6.

### Training

The PT-train data, containing 6787933 PSMs and 2550621 nonredundant peptide/*z*/*e* combinations (precursors), and the multistep training approach described in Methods were used to train FastSpel. The ProteomeTools data (both PT-train and PT-test) cover 24 peptide lengths (from 7 to 30), 6 charges (1 through 6), and 6 collision energies (20, 23, 25, 28, 30, and 35), which make overall 864 possible possible *l*/*z*/*e* combinations. However, for 514 of these combinations, the number of nonredundant peptides in PT-train did not satisfy our criterion ( $r_{lze}={Ñ}_{lzs}/\left(ml\right)\geq 1$ , with *m* = 21; see Methods). In fact, ${Ñ}_{lzs}=0$ for 247 out of the 864 possible *l*/*z*/*e* triplets. Thus, we could train FastSpel for only 350 combinations. This number (350) appears to be low, but one should keep in mind that the rest of combinations account for only 0.7% of PSMs in PT-train (and PT-test), and that FastSpel was trained, for at least one collision energy, for the most frequently occurring *l*/*z* combinations that are (*z* = 2, 7 ≤ *l* ≤ 24), (*z* = 3, 8 ≤ *l* ≤ 30), and (*z* = 4, 12 ≤ *l* ≤ 30). It is also worth noting that other intensity prediction methods also have limitations in this regard. For example, all precursors with *z* > 4 are automatically filtered out when running PeptDeep via Oktoberfest.

The list of the 350 *l*/*z*/*e* triplets for which FastSpel was trained, as well as the corresponding numbers of training steps are given in Table S1. Ranging from 2 to 15 with a median of 6, the reported numbers of steps suggest that the multistep training procedure improved the performance of FastSpel for all *l*/*z*/*e* combinations. Since the multistep approach was devised to eliminate small predicted peaks (increase the number of zeros in the predicted intensity profiles), we wondered how the predicted and observed intensity vectors compared in terms of the number of zero elements. For this purpose we used PT-test data and found that 74.0% of all predicted intensities vanished whereas this percentage for the corresponding observed intensities was 62.9%. On the other hand, 93.1% of the nonzero predicted peaks were also observed, while 65.4% of the observed peaks had corresponding nonzero predicted values. In other words, when using FastSpel, the nonzero predicted peaks are highly likely to be observed experimentally, but FastSpel misses some of the experimentally observed peaks. However, our results suggest that FastSpel mostly misses smaller peaks that are more likely to be noisy. Specifically, for PT-test data, we found that the observed peaks with zero corresponding predicted intensities had a mean intensity of 0.045. In comparison, the observed peaks that had corresponding nonzero predicted values were, on average, much larger with a mean intensity of 0.277. (Note that each ProteomeTools intensity profile has been normalized to have a maximum of 1). The distributions of the intensities of the observed peaks with zero and nonzero corresponding predicted peaks are shown in Figure S3.

PT-test data were also used to assess the effect of *r*_*lze*_ on the quality of predictions in terms of the cosine of the angle between the predicted and experimental intensity profiles. For each *l*/*z*/*e* combination for which FastSpel was trained, the median cosine, denoted by μ_*lze*_^*PT*^(test), calculated for the PT-test data as well as the corresponding median μ_*lze*_^*PT*^(train) for the PT-train data, the difference Δ_*lze*_ = μ_*lze*_^*PT*^(train) – μ_*lze*_^*PT*^(test), and the ratio *r*_*lze*_ are given in Table S1. The calculated μ_*lze*_^*PT*^(test) values are also plotted in Figure 1 A as a function of *r*_*lze*_. Figure 1 A shows that, generally, μ_*lze*_^*PT*^(test) increases with *r*_*lze*_, although there is an inflection point (at roughly *r*_*lze*_ = 10) after which the increase is much slower. This inflection point may provide an estimate of how much training data is good to have, but in practice for most *r*_*lze*_ values this much training data are not available. Moreover, Figure 1 A indicates high μ_*lze*_^*PT*^(test) values for many points before reaching (to the left of) the inflection point. Thus, the inflection point should *not* be considered as a cutoff below which training fails.

**Figure 1:**
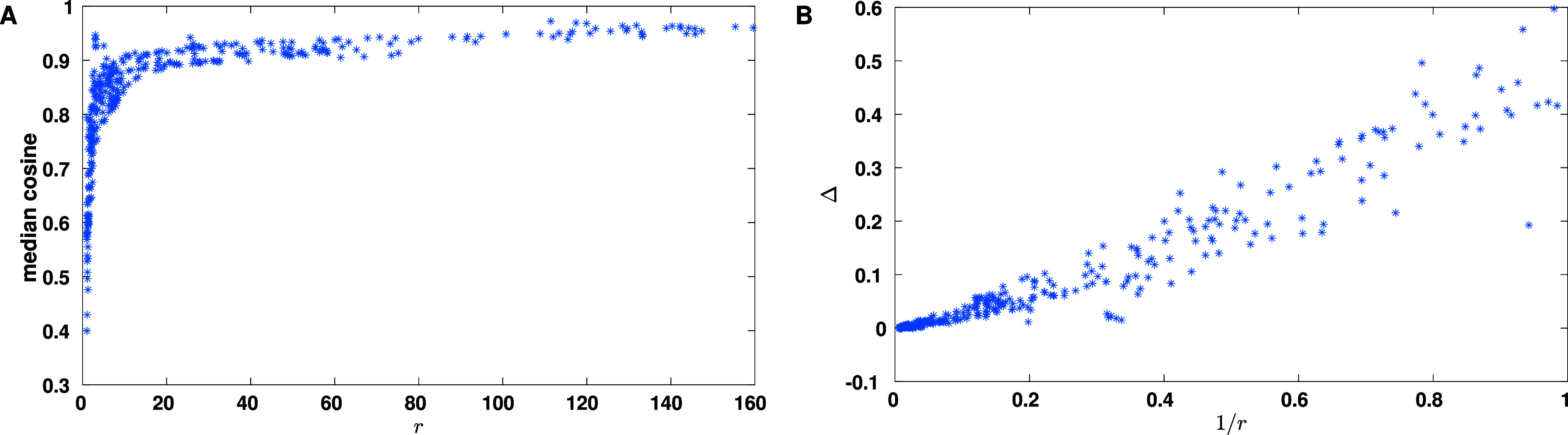
Effect of *r*_*lze*_ on training. (A) The median μ_*lze*_^*PT*^(test) is plotted as a function of $r_{lze}={Ñ}_{lzs}/M_{l}$ . (B) The difference between the medians calculated for PT-train and PT-test, that is Δ_*lze*_ = μ_*lze*_^*PT*^(train) – μ_*lze*_^*PT*^(test), is plotted as a function of 1/*r*_*lze*_.

To check for overfitting, we compared the two overall medians μ^*PT*^(test) and μ^*PT*^(train), calculated respectively over all PSMs in PT-test and PT-train (excluding PSMs associated with combinations for which FastSpel was not trained). We found close medians (μ^*PT*^(train) = 0.933 and μ^*PT*^(test) = 0.925), suggesting that overall FastSpel was not overfitted. However, we observed large differences Δ_*lze*_ = μ_*lze*_^*PT*^(train) – μ_*lze*_^*PT*^(test) for some individual *l*/*z*/*e* combinations. These differences are plotted as a function of 1/*r*_*lze*_ in Figure 1 B, which indicates large values for Δ in some cases, but shows that for most *l*/*z*/*e* combinations Δ_*lze*_ < 0.05. (This may not be obvious from the figure as many points with Δ_*lze*_ < 0.05 overlap; see Table S1). In fact, in PT-train only 7.7% of the PSMs belong to combinations with Δ_*lze*_ ≥ 0.05, which is the reason why the overall medians (μ^*PT*^(train) = 0.933 and μ^*PT*^(test) = 0.925) are so close.

In dealing with the issue of overfitting, one may choose a cutoff Δ_max_ and limit the application of FastSpel to *l*/*z*/*e* combinations for which Δ_*lze*_ < Δ_max_. However, any such a cutoff (or any cutoff based on Figure 1A) would be arbitrary. Furthermore, only a small percentage of precursors are associated with combinations that have large Δ*s*. Hence, we decided not to apply such a cutoff and use FastSpel for all combinations for which it was trained. Of note, overfitting arises because of lack of enough training data. Figure 1 and Table S1 suggest that if enough data are available, overfitting is avoidable and that the resulting trained method performs well. As shown later, FastSpel is fast to train, and so it can be quickly retrained if additional data become available.

Since FastSpel was not trained for some *l*/*z*/*e* combinations (associated with a tiny fraction of precursors), in all subsequent analyses reported in this study, the similarity measures α and *R* as well as the predicted intensities corresponding to these *l*/*z*/*e* values were all considered to be zero. In other words, we did not exclude any *l*/*z*/*e* combinations based on the results reported in this subsection. However, because PeptDeep (run using Oktoberfest) automatically filters out any peptide with *z* > 4, the following analyses were performed for PSMs with 7 ≤ *l* ≤ 30 and 1 ≤ *z* ≤ 4.

### Interpretation of Model Parameters

As mentioned previously, the elements of a column of *X*_*lze*_ can be regarded as the contributions of different amino acid/position combinations to the predicted intensity of the ion corresponding to the column (see the “Prediction” subsection of Methods and Text S1). Hence, by examining the elements of *X*_*lze*_, one may uncover patterns that may provide insights into fragmentation chemistry. In this subsection we identify a few most prominent such patterns.

Given the fact that there are a large number of matrices, each having a large number of elements, a deep dive into the interpretation of all parameters is beyond the scope of this paper and deserves a separate study. Thus, we limit this analysis to the case of *z* = 2, *e* = 28, and consider only singly charged fragments. Moreover, to include only the most reliably computed matrices, only peptides of length 7 to 20 are considered (for these cases a large number of training peptides were available with *r*_*lze*_ > 10; see Table S1 and the previous subsection). Additionally, only amino acid/position combinations with the largest contributions are considered. Since tryptic peptides almost always end in K or R, only these amino acids are considered in the last position (C terminus).

For each theoretically possible ion, Table S2 lists the amino acid/position combinations with the largest contributions to the intensity of the ion for the considered peptide lengths. These amino acid/position combinations are identified by finding the maximum value of each column of each *X*_*lze*_. Despite having a large number of entries, Table S2 shows some clear patterns that are briefly discussed here.

The most obvious pattern is proline favoring *y*_*i*_ ions, for *i* ≥ 3, in 120 out of the 147 cases listed in Table S2. In 119 of these 120 case, proline is the strongest promoter of *y*_*l*–*i*+1_ if it is in the *i*th position in the sequence. In other words, proline promotes *y* ions via cleavage at its N-terminal side, which has been reported in the literature as the “proline effect”.^25^ Interestingly, proline is not the largest contributor to *y*_1_ and *y*_2_ ions. For *y*_2_ ions, regardless of peptide length, this role is played by aspartic acid in position *l* – 2, an observation previously reported in some synthetic peptides.^26^ On the other hand, the largest contribution to *y*_1_ comes from an arginine in the first half of the sequence.

Table S2 also indicates that the strongest promoters of *b* ions are almost exclusively the basic residues arginine, histidine, and lysine positioned at some point before the cleavage site. This is not surprising as the basic residues tend to retain the charge. However, the three amino acids act differently depending on their positions in the sequence and on the ion length. Lysine, regardless of peptide length, is the largest contributor to *b*_1_ (when it is in the first position), but it rarely appears anywhere else in the table. For some sequence lengths, histidine favors *b*_*i*_ but only when located at the *i*th or (*i* – 1)­th position and only for 2 ≤ *i* ≤ 9. This suggests that, in some peptides, histidine favors *b* ions via cleavage at its C-terminal side, which has been also observed in experiments concerning doubly charged peptides.^27^ Arginine, on the other hand, is the strongest booster of *b*_*i*_ for some peptide lengths if 4 ≤ *i* ≤ 9, and regardless of length when *i* > 9. The position in which arginine serves as the most effective fragmentation promoter varies, but is usually closer to the beginning of the sequence, especially for shorter *b* ions. Table S2 suggests that the only nonbasic amino acid making the largest contribution to *b* ions is isoleucine. Specifically, for some peptide lengths, isoleucine favors *b*_3_ and *b*_4_ ions via cleavage at its N-terminal side. We could not find studies confirming the role of isoleucine and some of the other observations mentioned above (for example, arginine promoting *y*_1_ when located in the first half of the sequence). However, lack of evidence makes these new fragmentation patterns even more interesting as the may provide new insights into this subject.

It is important to note that the strength of a peak is dependent on the contributions from all residues in the peptide. Table S2 only lists the strongest contributions, but other weaker (or negative) contributions may significantly affect the observed intensities. Thus, the presence of the aforementioned most effective amino acids in the sequence does not necessarily mean certain ions will have large intensities.

### Performance Evaluation: Similarity

In this subsection we evaluate FastSpel, Prosit, PeptDeep, and MS2PIP in terms of the normalized angle α, correlation *R*, overlap *f*, and the product α*f*, which is one our candidates for the new scoring function (see Methods for details). These two similarity measures (*f*, α*f*) are considered here for two reasons: (1) to show examples of how the performance of a method could be measure-dependent, and (2) to include one of our candidate new scoring functions. We do not include the other candidate score (α*n*_op_, with *n*_op_ being the number of matches) because there is no theoretical optimal value for this score to compare to.

To make these evaluations, we used the four methods to predict the intensity profiles for the PSMs identified by MaxQuant, at the FDR = 1% level, for the 22 DDA testing data sets. These predicted intensities were then compared with the corresponding experimental profiles. Of note, since PeptDeep (MS2PIP) does not predict triply (triply and doubly) charged fragment intensities, to have a fair comparison, only singly charged fragments were considered for this analysis. However, when rescoring peptides all predicted intensities were included as in this case no similarity measure comparison was made between the methods.

Figure 2 shows box plots of the four aforementioned similarity measures. To generate the figure, the 22 testing data sets were partitioned into two groups based on the instrument type (QE and Fusion), and then the similarity measures corresponding to the PSMs in each group were combined into one set (7241291 QE and 2250234 Fusion PSMs). We separated the QE and Fusion data because the performance of the methods may be instrument-dependent.^12,16^ On the other hand, for each instrument type and each similarity measure, the data points were combined to present an overall picture. For individual data sets, the medians and quartiles of the similarity measures and the number of PSMs are given in Table S3.

**Figure 2:**
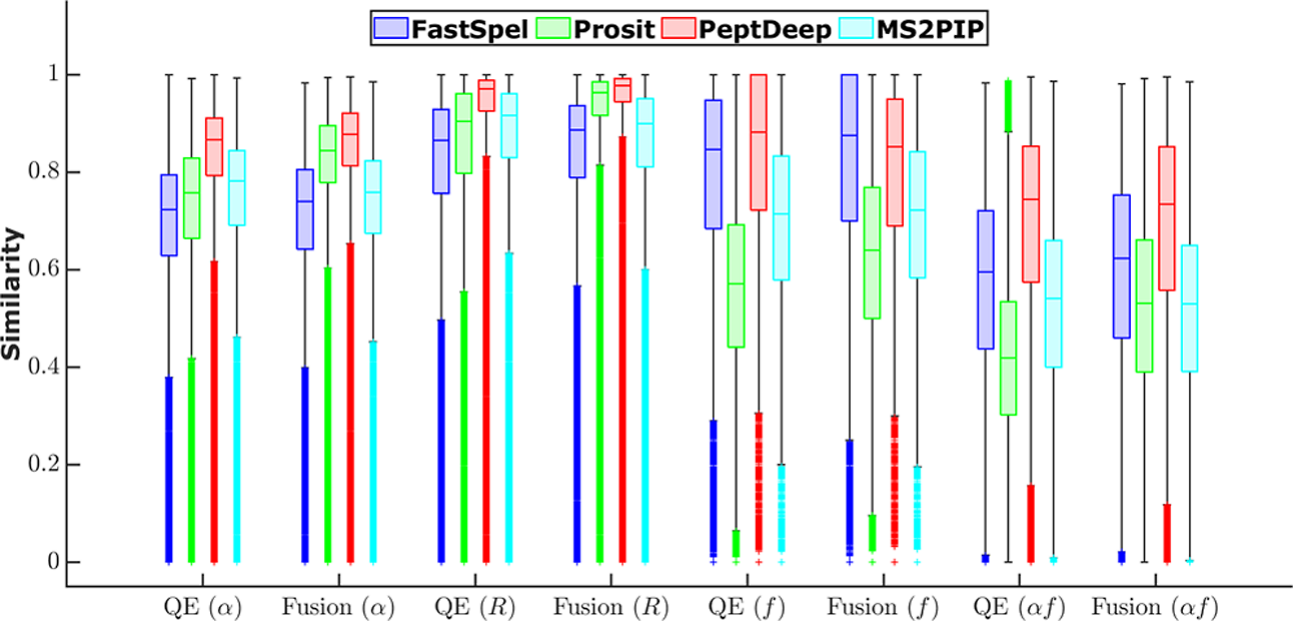
Boxplots of similarity measures. Boxplots of normalized angle (α), correlation (*R*), overlap (*f*), and overlap multiplied by normalized angle (α*f*) are shown separately for QE and Fusion instruments for the four evaluated methods. The outlier points are shown using “+” markers, but since there are many of them and they are overlapping they look like continuous lines below the lower whisker. Of note, since the intensities are non-negative, angles are non-negative as well. Correlations, on the other hand, can be negative and indeed the tails of the distributions go below zero. However, for clarity, negative parts of the tails of the correlation plots are not shown here.

As expected, Figure 2 demonstrates similar trends for α and *R*, that is better performance by the more sophisticated methods. However, FastSpel is not far behind. In agreement with other reports,^12,16^ the figure indicates that Prosit performs better when applied to Fusion/HCD (as opposed to QE/HCD) data. This is presumably because Prosit was trained on ProteomeTools data that were obtained using Fusion instruments. Interestingly, although FastSpel was also trained on only Fusion data, the drop in QE vs Fusion median similarity in our case is smaller than that of Prosit, suggesting that FastSpel is less sensitive to changing the instrument type.

An interesting observation from the figure is that, regardless of the prediction method, the distributions have long tails (many outliers). Since we did not filter the identified peptides based on the Andromeda score or any other criteria, some of the experimental profiles may be quite noisy, resulting in poor similarity measures. Misidentified peptides (false discovery rate of 1%) are also likely to have contributed to the elongation of the tails.

When comparing the methods based on the other two similarity measures (*f* and α*f*), the situation is different. When evaluated based on *f* or α*f*, FastSpel beats Prosit and MS2PIP. On the other hand, FastSpel and PeptDeep perform comparably (slightly better/worse for QE/Fusion instrument types) when assessed using the overlap *f*. The fact that FastSpel performs well in this case is not surprising. As mentioned previously, because of the multistep training procedure, the peaks predicted by FastSpel are highly likely to be observed.

It should be noted that in the case of Prosit, collision energy calibration is recommended for QE/HCD data and has been reported to improve the normalized angles/correlations.^12^ However, collision energy calibration requires known PSMs. In this analysis the known PSMs are used for assessment, and since the same PSMs cannot be used for both calibration and evaluation, collision energy calibration was not performed in this case. One can set aside a portion of the data for calibration and test the calibrated method with the rest of the data. However, since our main point is to compare the methods based on their performance in separating true targets from decoys and false targets, we investigate the effect of collision energy calibration in the following subsection.

One factor that could potentially affect the performance of the methods is the possible overlap between the peptides used for training and those used for testing the methods. We investigated the overlap between our training data (PT-train) and testing (the 22 DDA data sets) and found the overlap to be small (3% overall overlap when the 22 data sets are combined, and 6.4% maximum overlap for PXD021588). Moreover, PT-train data have been also used for training Prosit and PeptDeep, and so any overlap is expected to affect the three methods (FastSpel, Prosit, and PeptDeep) in the same way. Hence, we conclude that the overlap does not significantly affect the results presented here.

### Performance Evaluation: Rescoring and Peptide Identification

In this subsection we make comparisons (1) between the four prediction methods, and (2) between several scoring functions, in terms of the number of identified peptides after rescoring. To make these comparisons, all PSMs (targets/decoys) identified by MaxQuant (FDR = 100%) in the 22 DDA testing data sets were rescored using different scoring functions and Percolator (under different scenarios; see Methods) with the features computed using the four prediction methods, both with and without collision energy calibration.

It is worth mentioning that collision energy calibration is usually a part of the rescoring process. Specifically, one cannot turn off collision energy calibration in Oktoberfest. However, when methods are used for spectral library generation, identified PSMs may not be available for collision energy calibration. For this reason, in this subsection we first compare the uncalibrated methods, and then investigate the effect of collision energy calibration.

#### Uncalibrated Methods

The total (summed over the 22 data sets) numbers of peptides identified by five scoring functions and Percolator, run with two different sets of features, are shown in Figure 3A,B for QE and Fusion instruments, respectively. The numbers of identified peptides for individual data sets are given in Table S4. The five scoring functions are normalized angle (α), overlap (*f* = *n*_op_/*n*_p_), number of matches (*n*_op_), and our two candidate scoring functions, namely α*f* and α*n*_op_. The two Percolator runs included intensity-related plus general features (IG), and all features (IGRTC; full rescoring pipeline with collision energy calibration). Note that Percolator (IGRTC) could not be run for FastSpel because FastSpel does not predict retention times. Nonetheless, for the other three methods the results of Percolator (IGRTC), which is expected to identify the largest number of peptides, are shown as a reference only. (We could not run Percolator (IGRT) without calibration).

**Figure 3:**
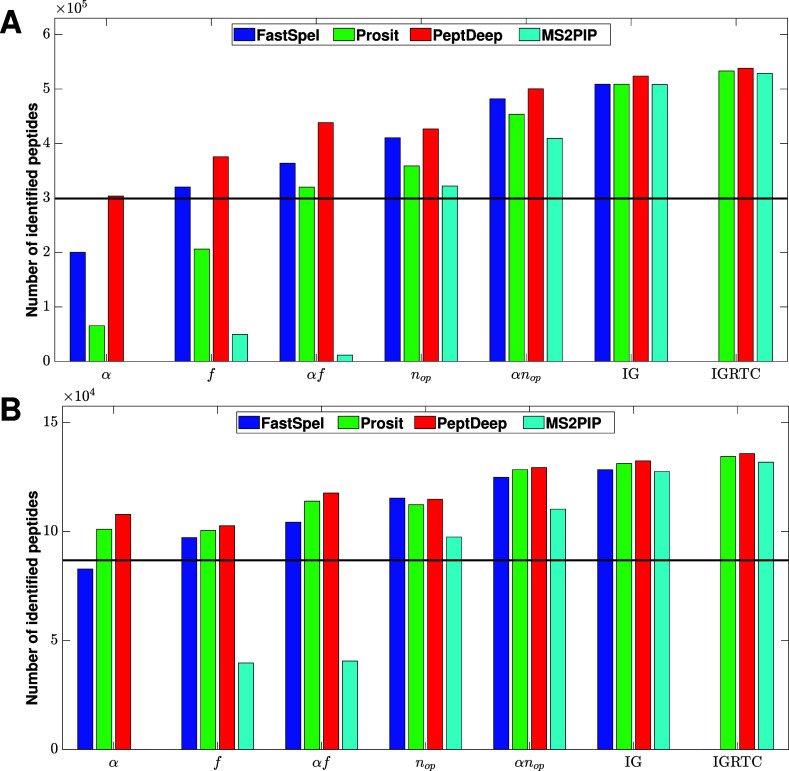
Number of identified peptides. The total number of peptides identified using different rescoring methods/functions and various prediction approaches are plotted for (A) QE and (B) Fusion instruments. Here, α, *n*_op_, and *f* = *n*_op_/*n*_p_ respectively denote normalized angle, number of matches (peaks that are both observed and predicted), and overlap that is the fraction of predicted peaks that are also observed. IG, and IGRTC respectively denote Percolator results obtained using intensity-related plus general features, and using all features including retention time-related features. Except for the case of Percolator (IGRTC), all intensity-related features were computed employing the uncalibrated methods. MS2PIP failed to identify any peptides using α as score, and so the corresponding bar is missing. Currently, FastSpel does not have the capability to predict retention times, and so Percolator (IGRTC) results are shown for the other three methods. The horizontal line shows the number of peptides identified using the Andromeda score.

Despite some differences (for example, overlap being better than normalized angle for QE instruments), Figure 3 A and B show similar trends and suggest that

- In comparison with other scoring functions and Percolator, normalized angle does not perform well and in some cases is even worse than the Andromeda score (the horizontal black line in the figure). Interestingly, the figure indicates no peptides were identified using α computed by MS2PIP, an observation also reported by Gessulat et al.^8^ This demonstrates that higher α between predicted and observed intensities of true targets, shown in Figure 2, does not necessarily translate to better peptide identification.
- The number of matches (*n*_op_) by itself performs pretty well (better than α and *f*) as a scoring function. This is not surprising as getting a high *n*_op_ by chance is not likely.
- α*n*_op_ performs well and is the best among the simple scoring functions.
- For FastSpel and PeptDeep (and Prosit/Fusion), α*n*_op_ performs almost as well as Percolator (IG) and even Percolator (IGRTC), especially in the case of Fusion instrument type. The ratios of the numbers of peptides identified by α*n*_op_ to those identified by Percolator (IG) are 0.97, 0.98, 0.95, and 0.96 for, respectively, FastSpel/Fusion, PeptDeep/Fusion, FastSpel/QE, and PeptDeep/QE. In the case of Percolator (IGRTC) these ratios are 0.96 and 0.93 for PeptDeep/Fusion and PeptDeep/QE respectively. Thus, we propose α*n*_op_ as our new scoring function
- Most importantly, based on the best performing rescoring function/method, that is Percolator (IG), FastSpel performs comparably with the other three prediction methods. Specifically, the ratio of the numbers of peptides identified by FastSpel and PeptDeep, which performs a tiny bit better than the others, is around 0.97 for both QE and Fusion instrument types. Additionally, FastSpel performs comparably with PeptDeep when PSMs are rescored using α*n*_op_.

It is important to point out that the last two observations itemized above imply that the good performance of FastSpel + Percolator (IG) is *not* due to the presence of the general features or running Percolator. FastSpel + Percolator (IG) performs well because of the high quality of the predicted intensities, which is evidenced by the comparable numbers of peptides identified by FastSpel and PeptDeep using α*n*_op_ as the scoring function. For both FastSpel and PeptDeep, adding the general features to the intensity-related features and running Percolator only slightly improves peptide identification in comparison with using only α*n*_op_ as the rescoring function.

To explain how a method as simple as FastSpel can perform comparably, in terms of the number of identified peptides, with sophisticated methods like PeptDeep, one can look for clues in the proposed scoring function α*n*_op_. The performance of α*n*_op_, especially when calculated using FastSpel or PeptDeep, is impressive given that it uses the product of only two intensity-related features (as opposed to tens of intensity-related features used by Percolator) and does not use any of the general or retention time-related features. Moreover, Percolator trains a model for separating targets from decoy using (a portion of) the data. We attribute the good performance of α*n*_op_ to the fact that it combines two types of information: how well the magnitudes of intensities agree with each other (α) and how many matches are there (*n*_op_). Although all elements in the intensity vectors (including all matched peaks) affect α, small peaks, especially the ones that are small in both predicted and observed profiles, contribute less to α. However, the discriminating power of *n*_op_ (Figure 3) suggests that matched small peaks may play a role in separating true targets from false ones and decoys. In fact, Figure 3 suggests that *n*_op_ contributes more to the discriminating power of α*n*_op_ than α does. This view is supported by the fact that the vast majority of the intensity-related features used by Percolator are calculated based on the presence/absence of the peaks (nonzero vs zero) rather than the magnitude of the nonzero peaks. It is presumably easier to predict the presence/absence of the peaks (for example by the multistep training procedure described in Methods) than to predict their magnitudes. This is perhaps one of the reasons why FastSpel is successful despite predicting “less accurate” intensity profiles.

It should be emphasized that although FastSpel does not perform as well as other models in terms of similarity (measured by α) between the predicted and observed intensities, it comes close to the better performing models (Figure 2). Without this level of accuracy, FastSpel would not have been as good, because multiplication of *n*_op_ by α does improve the results for all methods (Figure 3). Thus, one should not deduce from our results that improving prediction accuracy is not important for rescoring. When/if a significantly better prediction method is developed, the rescoring results are likely to improve, and α by itself may become a reliable scoring function.

Another reason for the good performance of FastSpel is that it is trained for specific values of *l*/*z*/*e*. In comparison with other methods, FastSpel is very simple, but it compensates for its simplicity by effectively training hundreds of separate models, each of which require pretty large amount of data to produce good results (Figure 1). This attribute is also the source of the some of the current limitations of FastSpel as discussed in the FastSpel Limitations subsection.

#### Effect of Collision Energy Calibration

The total numbers of identified peptides obtained using scores/features computed by the collision energy calibrated versions of the methods are shown in Figures S4A,B for QE and Fusion instruments respectively. The numbers obtained for individual data sets are given in Table S5. Despite some differences, for example better performance of α, trends seen in Figure S4 are similar to the ones shown in Figure 3, and the observations itemized in the previous subsection are still valid for the calibrated case. Comparing Figures 3 and S4, no large changes are observed in the number of peptides identified by Percolator (IG vs IGC) or by the proposed score α*n*_op_. However, in some cases modest improvements are observed due to calibration. Specifically, for QE data, the figures indicate 10.0%, 2.3% and 2.6% improvement in the numbers of peptides identified by, respectively, Prosit/α*n*_op_, Prosit/Percolator, and FastSpel/α*n*_op_. In the rest of the cases improvements are less than 1%.

#### Overlap between Peptides Identified Using Different Methods/Scores

Next, we investigated the overlap between the peptides identified by different methods. The results, shown in Figure 4, show high overlap between the identified peptides. For example, more than 98% of the peptides identified by FastSpel + Percolator are also identified by PeptDeep + Percolator. Of note, Figure 4 shows the total numbers of peptides identified by the uncalibrated methods for all 22 data sets, regardless of the instrument type. Since, we concluded that calibration does not have a significant effect, we limited our investigation to the uncalibrated methods. Also, since for both QE and Fusion instrument types there were no significant differences between the methods, in terms of the number of peptides identified by Percolator, for brevity we have combined the results for the two instrument types. We also investigated, for each prediction method separately, the overlap between the peptides identified by α*n*_op_ and those found by Percolator. The result, shown in Figures S5–S8, again show high overlap between the two sets of peptides. For example, in the case of FastSpel, more than 98% of the peptides identified by α*n*_op_ are also identified by Percolator.

**Figure 4:**
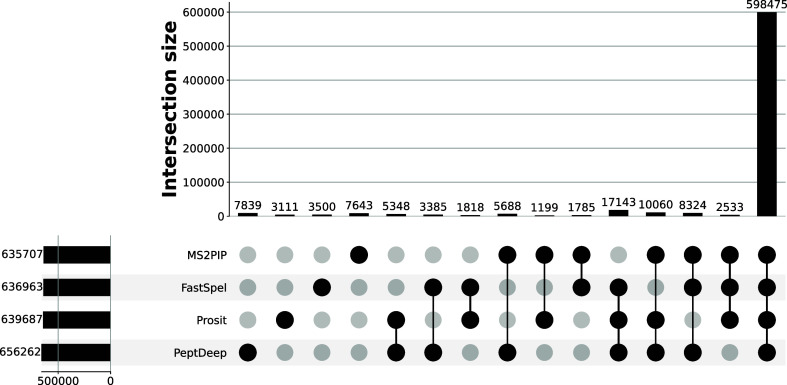
Overlap between the identified peptides. The UpSet plot compares the sets of peptides identified by Percolator (IG), when different methods are used to compute the intensity-related features. The horizontal bars (lower left) show the total numbers of identified peptides by each method. The vertical bars show the numbers of peptides exclusively identified by each of the methods or by any combination of the 4 methods. Peptides exclusively identified by 2 methods, for example, are the ones identified by both of these methods, but missed by the other methods. The (connected) black dot(s) below each vertical bar indicate(s) which method(s) were used to calculate the number of peptides represented by the bar. The last bar (corresponding to the intersection of peptides identified by all methods) is very large compared with the others, indicating the 4 methods essentially identify the same peptides.

### Performance Evaluation: DIA Analysis

DIA search was performed using spectral libraries generated by the four methods and employing DIA-NN for the data set PXD028735, a three species (human, yeast, and *E. coli*) benchmark data set with known relative protein abundances under three different conditions. The numbers of identified peptides (proteins) were respectively 76646, 79957, 85037, and 85373 (10281, 10630, 10902, and 10930) for MS2PIP/DIA-NN, FastSpel/DIA-NN, Prosit/DIA-NN, and PeptDeep/DIA-NN, indicating comparable performances, especially in terms of the number of identified proteins, with PeptDeep being only a bit better. Moreover, high overlaps were observed between the peptides (Figure S9), and proteins (Figure S10), identified by the four methods, with 96% (94%) of peptides (proteins) identified by FastSpel/DIA-NN, also identified by PeptDeep/DIA-NN. Additionally, the methods performed comparably in terms of protein quantification. Specifically, for each species, method, and pair of conditions, the median log fold change was compared with the corresponding experimentally known logarithm of relative protein abundance, and the error was calculated as the absolute value of the difference between the computed median and the expected (known) value. The mean errors, averaged over all pairwise conditions for all three species, were 0.075, 0.079, 0.093, and 0.088 for FastSpel/DIA-NN, MS2PIP/DIA-NN, Prosit/DIA-NN, and PeptDeep/DIA-NN, respectively. The results indicate small and comparable errors for the four methods, with FastSpel having a slightly lower error. The distributions of log fold changes for *E. coli*, yeast, and human are shown in Figures S11–S13, respectively.

### Performance Evaluation: Prediction Speed

Run times were compared in three different ways: (1) “apple to apple” comparisons between the relative prediction times when methods are run on the same system, (2) “practical” comparisons by running Prosit/PeptDeep/MS2PIP on a GPU using Koina^28^ (the public server used by Oktoberfest), and running FastSpel on a single CPU, and (3) comparisons between the relative total times when using the same CPUs. Here, we first present the results of these comparisons and then discuss the time needed for training FastSpel. To run PeptDeep and MS2PIP on CPUs, we used their respective Python packages, whereas Prosit was run through the inSPIRE.^29^ The details regarding these runs and estimation of run times are given in Text S7.

#### Running the Methods on the Same System

CPU prediction time comparisons were made using 1, 8, 16, and 32 CPUs. For each method, the relative prediction time, that is the prediction time of the method divided by that of FastSpel, was then estimated (for details see Text S7). The mean relative prediction times (averaged over the 22 DDA testing data sets) for Prosit, PeptDeep, and MS2PIP are shown in Figure 5. Note that, since the times are relative, the prediction time of FastSpel is always 1, and thus is not plotted in the figure. The actual prediction times for each of data sets as well as the number of precursors, are given in Table S7. Figure 5 indicates that, using only 1 CPU, FastSpel is hundreds of times faster than the other three methods. Increasing the number of CPUs reduces the prediction times. However, as expected, the speedup decreases as the number of CPUs increases. For example, going from 16 to 32 CPUs, the average relative times decrease by only 15%, 21%, and 23% for Prosit, PeptDeep and MS2PIP respectively. And even with 32 CPUs the three methods are tens of times slower than FastSpel. The drop in speedup is expected to grow to the point that no further improvement is observed when the number of CPUs is increased. Given the high computational cost, we did not run the methods using more than 32 CPUs. However, if we assume that when doubling the number of CPUs the relative prediction times continue to drop by the same factors as they do when the number of CPUs is changed from 16 to 32, using even 1000 CPUs all three methods will still be at least 10 times slower than FastSpel. On the other hand, although employing more CPUs (up to a point) decreases the prediction times, the computational cost, measured by CPU seconds, actually increases. We thus conclude that FastSpel is over 2 orders of magnitude less computationally expensive and more than 1 order of magnitude faster than the three other methods regardless of the number of CPUs used.

**Figure 5:**
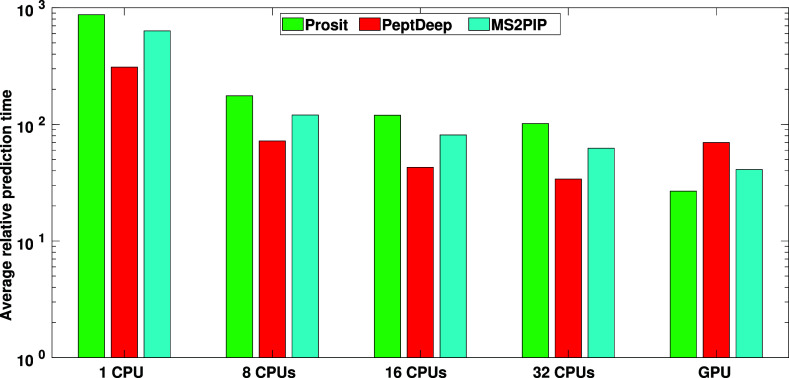
Average relative prediction time. The average relative prediction times are plotted for Prosit, PeptDeep, and MS2PIP. Relative time for each method is defined as *t*(method)/*t*(FasSpel), where *t*(method) and *t*(FasSpel) are respectively the time spent by the method and FasSpel on prediction only (excluding time spent on input/output). In all cases, including GPU, *t*(FasSpel) is the time FastSpel spent using only one CPU. Since relative time for FastSpel is 1, it is not shown in the figure. Note that the time axis is in the logarithmic scale.

#### Practical Runtime Comparison

Using the Koina server (GPU) the Prosit/PeptDeep/MS2PIP average relative prediction times decrease dramatically (compared with the corresponding runs on one CPU), but they are still more than 1 order of magnitude slower than FastSpel (Figure 5 and Table S7). One should keep in mind that the times measured for these “practical comparisons” were divided by the corresponding 1-CPU time of FastSpel to get the relative times. Obviously, the measured times in this case are dependent on the specifics of the CPU employed for making the predictions. Thus, the corresponding GPU and CPU times are not exactly comparable. Using different CPUs may change the relative times reported here. However, these changes are unlikely to invalidate our conclusion that a typical Prosit/PeptDeep/MS2PIP run on the Koina server is about 1 order of magnitude slower than a typical FastSpel run on a CPU. An interesting observation form Figure 5 is that in the GPU runs Prosit is the fastest and PeptDeep is the slowest method. When using CPUs, however, the opposite is true. It is not clear to the authors why this is the case, but perhaps the fact that Prosit was specifically designed to work with GPUs contributes to this difference.

#### Total Runtime Comparison

To have a complete investigation, we also compared the total relative times, that are the total times spent on reading/predicting/writing divided by the corresponding FastSpel times. The average relative times using 1, 8, 16, and 32 CPUs are shown in Figure S14 and the actual times for individual data sets are given in Table S8. The results (Figure S14) again indicate that FastSpel is between 1 and 2 orders of magnitude faster than the other three methods.

It is important to note that prediction of intensities for target and decoy PSMs is only a part of the rescoring process. Hence, reduction of the time spent on the whole rescoring pipeline is not going to be as dramatic as the ones shown in Figures 5 and S14. Nonetheless, given that FastSpel performs comparably with other methods, even a modest decrease in the run time may be beneficial. More importantly, although we used rescoring in this study to evaluate the quality of the predicted intensities, the main goal of FastSpel is fast generation of spectral libraries, which may have applications other than rescoring. For example, FastSpel in conjunction with our proposed scoring function α*n*_op_, which we have shown outperforms the Andromeda score, may be used for on-the-fly scoring of PSMs in the initial search by programs like MaxQuant, potentially improving the initial (before rescoring) search results. Of course, the potential application of FastSpel for such a purpose must be investigated rigorously and is beyond the scope of this paper.

#### Training Time

Using 8 CPUs, FastSpel took 226 s to train (AMD EPYC 3.8 GHZ CPUS; training time only, excluding the time spent on reading the data). This amounts to less than 40 s per collision energy. It is therefore quite fast to retrain FastSpel with more training data, or to train it for more collision energy values, new instruments, CID data, or nontryptic peptides.

### FastSpel Limitations

FastSpel has three main limitations in comparison with other prediction methods: (1) no retention time/ion mobility prediction capability, (2) intensity prediction only for tryptic/HCD data, and (3) predictions limited to only *b*/*y* ions of peptides that are unmodified or have only oxidized methoinines as variable modifications. Although in principle an approach similar to that of FastSpel can be used to predict retention time and ion mobility, the accuracy of such an approach needs rigorous testing and comparison with other methods, and so is beyond the scope of this manuscript. However, it should be pointed out that limitation (1) is not really related to intensity prediction. Spectral libraries can be generated without retention time prediction, which is independent of intensity prediction. Additionally, we have shown that, at least for tryptic peptides, including the retention time-related features in the input of Percolator only slightly improves peptide identification (Figure 3). Moreover, if/when needed, one may utilize FastSpel in conjunction with a retention time prediction method. In fact, MS2PIP by itself does not predict retention times; instead it uses DeepLC.^22^ Based on these observations, we believe lack of retention time prediction capability does not hinder usage of FastSpel.

Limitation number (2) is potentially more important. FastSpel has been only trained for tryptic/HCD data. However, this does not mean that it cannot be trained for cases with nontryptic peptides, CID data, or trapped ion mobility time of flight mass spectrometer (timsTOF) data. Our results (Figure 1) suggests that a rather large amount data is needed for training FastSpel. The amount of data needed to train FastSpel for nontryptic data is vastly larger, because the number of possible peptides of given length is much bigger than that in the case of tryptic peptides. Nonetheless, given enough data, FastSpel can be quickly trained for nontryptic peptides and other situations. However, given the large amount of data needed for training, which may not be readily available, and the rigorous tests required to evaluate the method for each situation, training/testing FastSpel for other situations is beyond the scope of this paper. Instead, here we introduce FastSpel as a proof of concept that can be improved upon in the future. However, it should be emphasized that, even in its current form, FastSpel can be of practical use as the majority of mass spectrometry data are obtained using trypsin digestion and HCD fragmentation type.

The third limitation of FastSpel is predicting only *b*/*y* ion intensities and accepting only oxidation of methoinine as a variable modification. Although some of the previously proposed methods (e.g., Prosit) suffer from the same limitation, others are less limited in this regard. For example, PredFull^10^ has been proposed to predict the full spectrum and PeptDeep and MS2PIP accept peptides with other modifications. However, it should be emphasized that this limitation stems from the absence of other modifications and intensities of other ion types in our training data. Given enough training data, it is straightforward to retrain FastSpel to predict intensities of other ions and to handle various modifications.

## Conclusions

As a proof of concept, in this paper we introduce FastSpel, a fast, simple, and interpretable method for predicting high quality MS/MS peptide fragment intensities. Despite being slightly less accurate (measured by α) than the other methods, in terms of identifying peptides via rescoring, and peptide/protein identification/quantification via DIA searches, FastSpel performs comparably with the state-of-the-art methods such as Prosit and PeptDeep. The main advantages of FastSpel, however, are its low computational cost, and simplicity/interpretability. The computational cost of FastSpel is more than 2 orders of magnitude lower than those of Prosit, PeptDeep, and the simpler method MS2PIP. On the other hand, the parameters of FastSpel can be related to what we know about fragmentation chemistry, potentially providing insights into this subject. Currently, FastSpel is limited to tryptic/HCD data. However, given enough data, it can be quickly trained for other situations. Also, even in its current form FastSpel covers most experimental situations and can be helpful in reducing the computational cost of MS/MS intensity prediction.

In addition to FastSpel, a new scoring function is introduced. The proposed score, which is simply the product of the normalized angle and the number of matches between the corresponding predicted and observed peaks, performs nearly as well as Percolator in respect to the number of identified peptides after rescoring. Together, FastSpel and the proposed score may be helpful for on-the-fly scoring of PSMs in database search or de novo peptide sequencing. This potential, however, requires careful evaluation that is beyond the scope of this paper. In the future, we plan to assess FastSpel in this regard and to integrate it in our microorganism identification tool MiCId.^30,31^ FastSpel Python package is available on our Web site (https://www.ncbi.nlm.nih.gov/CBBresearch/Yu/downloads/fastspel.html).

## Supplementary Material





## Data Availability

In this study we have used publicly available data sets downloaded from the PRIDE repository (https://www.ebi.ac.uk/pride/). The accession number for the DIA data used in the study is PXD028735. The accession numbers for the 22 DDA data sets are given in Table 1.

## Notes

Table S1: Number of steps and other information related to training. Table S2: Amino acid/positions with largest contributions. Table S3: Similarity measures for each data set. Table S4: Number of identified peptides for each data set; uncalibrated methods. Table S5: Number of identified peptides for each data set; calibrated methods. Table S6: CPU Prediction times. Table S7: GPU Prediction times. Table S8: CPU Total times (XLXS) (XLSX)
Text 1: Constructing the matrices *S*_*lze*_ and *I*_*lze*_. Text S2: Training details. Text S3: Number of linearly independent rows of *S*_*lze*_. Text S4: General solution to the minimization problem. Text S5: Running Percolator. Text S6: Summary of symbols. Text S7: Prediction speed estimation. Figure S1: Construction of a sequence matrix. Figure S2: Training workflow. Figure S3: Distribution of the intensities of observed peaks. Figure S4: Number of peptides identified by the calibrated prediction methods. Figure S5: Overlap between the identified peptides (FastSpel). Figure S6: Overlap between the identified peptides (Prosit). Figure S7: Overlap between the identified peptides (PeptDeep). Figure S8: Overlap between the identified peptides (MS2PIP). Figure S9: Overlap between peptides identified by DIA-NN. Figure S10: Overlap between peptides proteins by DIA-NN. Figure S11: Protein quantification comparison: *E. coli*. Figure S12: Protein quantification comparison: yeast Figure S13: Protein quantification comparison: human Figure S14: Relative total times (CPU) (PDF)
