# Supplementary material for: FastSpel: A Method for Fast Spectral Library Generation
Source: J Proteome Res. 2025 Aug 27;24(10):4952–64. doi: 10.1021/acs.jproteome.5c00279 (PMC12501936; doi:10.1021/acs.jproteome.5c00279)
Supplement: Supplementary file 2 [file pr5c00279_si_002.pdf]

Supporting Information
FastSpel: A method for fast spectral library generation

Mehdi B Hamaneh[1] and Yi-Kuo Yu[1*]

[1]Division of Intramural Research, National Library of Medicine, National Institutes of Health, Bethesda, MD 20894, USA
[*]Email: yyu@ncbi.nlm.nih.gov

Table of contents

- Text 1: Constructing the matrices S_{lze} and I_{lze}
- Text S2: Training details
- Text S3: Number of linearly independent rows of S_{lze}
- Text S4: General solution to the minimization problem
- Text S5: Running Percolator
- Text S6: Summary of symbols
- Text S7: Prediction speed estimation
- Figure S1: Construction of a sequence matrix
- Figure S2: Training workflow
- Figure S3: Distribution of the intensities of observed peaks
- Figure S4: Number of peptides identified by the calibrated prediction methods
- Figure S5: Overlap between the identified peptides (FastSpel)
- Figure S6: Overlap between the identified peptides (Prosit)
- Figure S7: Overlap between the identified peptides (PeptDeep)
- Figure S8: Overlap between the identified peptides (MS2PIP)

- Figure S9: Overlap between peptides identified by DIA-NN
- Figure S10: Overlap between peptides proteins by DIA-NN
- Figure S11: Protein quantification comparison: Ecoli
- Figure S12: Protein quantification comparison: Yeast
- Figure S13: Protein quantification comparison: Human
- Figure S14: Relative total times (CPU)
- Table S1: Number of steps and other information related to training (XLXS)
- Table S2: Amino acid/positions with largest contributions(XLXS)
- Table S3: Similarity measures for each dataset (XLXS)
- Table S4: Number of identified peptides for each dataset; uncalibrated methods (XLXS)
- Table S5: Number of identified peptides for each dataset; calibrated methods (XLXS)
- Table S6: CPU Prediction times (XLXS)
- Table S7: GPU Prediction times (XLXS)
- Table S8: CPU Total times (XLXS)

1 Supplementary texts

1.1 Text S1: Constructing the matrices S_{lze} and S_{lze}

Before explaining how a set of peptide sequences is represented by a matrix, we should emphasize that we consider only peptides that consist of a subset of the 20 prevalent amino acids plus oxidized methionine (overall $m = 21$ amino acids). Specifically, rare amino acids such as selenocysteine ("U") and variable modifications other than methionine oxidation are excluded. All cysteines are considered to be carbamidomethylated (fixed modification).

To represent a peptide sequence by a vector, the $m = 21$ amino acids are assigned the integers $1, 2, \ldots, m$. The amino acid with assigned integer j can be encoded by a $1 \times m$ vector whose elements are all zero except for the jth element, which is equal to 1 (one-hot encoding). The $M_l = lm$ dimensional vector representing the whole sequence of a peptide of length l is then obtained by horizontally stacking the $1 \times m$ vectors corresponding to the l amino acids in the sequence. Consequently, the sequences of a set of N_l peptides of length l is represented by an $N_l \times M_l$ matrix S_l, in which each row corresponds to a

peptide sequence. Figure S1 shows a simple example of how the matrix S_l is constructed.

The (predicted or observed) intensity profile of a peptide depends on its charge and sequence. Additionally, the collision energy used in the experiment affects the intensities. Hence, when constructing the matrices containing the intensities, one should be specific to a particular length, charge, and collision energy. For a set of N_{lze} peptides of length l and charge z, the b and y peak intensities for collision energy e are arranged in an $N_{lze} \times K_{lz}$ matrix I_{lze}, where each row contains the intensities corresponding to a peptide. Here $K_{lz} = 2(l-1)z_{\mathrm{f}_{\max}}$ is the number of possible peaks corresponding to fragments with charges up to $z_{\mathrm{f}_{\max}} = \min(3, z)$. (Fragment charge is denoted by z_{f} as opposed to z that denotes peptide charge.) In each row of I_{lze}, the intensities are arranged in the following order: $y_1^1, [y_1^2], [y_1^3], b_1^1, [b_1^2], [b_1^3], \ldots, y_{l-1}^1, [y_{l-1}^2], [y_{l-1}^3], b_{l-1}^1, [b_{l-1}^2], [b_{l-1}^3]$. Here the superscripts are the fragment charges, and the brackets indicate peaks that theoretically may not be present depending on the peptide charge (For example, a peptide of charge 1 cannot have doubly or triply charged fragments).

Note that the intensity matrices are constructed separately for different l, z, and e values. Accordingly, for each $l/z/e$ combination, we build a separate sequence matrix (denoted by S_{lze}), although the values of z and e have no effect in this case. The corresponding rows of I_{lze} and S_{lze} relate to the same PSM.

1.2 Text S2: Training details

Given some training data containing peptide sequences and their corresponding observed intensity profiles, for each $l/z/e$ combination, the sequence matrix S^t_{lze} and the intensity matrix I^t_{lze} are constructed (see the "Matrix representations and notation" subsection). Here the superscript ^{t} indicates that the matrices are generated using the training data. A multi-step approach is then taken to find the X_{lze} matrix. In the nth step, X^n_{lze} is found by minimizing

$$g_{lze}(n) = \|I^{nt}_{lze} - S^t_{lze} X^n_{lze}\|, \tag{S1}$$

where $\| \bullet \|$ denotes the Frobenius norm of the matrix $\bullet$, and I^{nt}_{lze} is the observed intensity matrix with its zero values replaced by $-(n-1)C$. Here $C > 0$ is a small constant, which in this study was set to 0.05. The rationale behind using a multi-step approach and replacing the zero elements in I^t_{lze} by $-(n-1)C$ is explained in the following paragraph.

In the first step $g_{lze}(1) = \|I^t_{lze} - S^t_{lze} X^1_{lze}\|$ is minimized to find X^1_{lze}. The filtered predicted intensities I^{1p}_{lze} are then calculated by replacing the negative values in $I^1_{lze} = S^t_{lze} X^1_{lze}$ by zeros, which further decreases the difference between the predicted and experimental intensities ($\|I^t_{lze} - I^{1p}_{lze}\| \leq \|I^t_{lze} - I^1_{lze}\|$). Although this replacement introduces zeros in I^{1p}_{lze}, there still may be many positive small predicted peaks with no corresponding observed peaks. Getting rid of these falsely predicted small peaks (having more zeros in I^{1p}_{lze}) may result in still more accurate predicted intensities. This is exactly why the next steps are taken. As mentioned previously, in the nth step, to get I^{nt}_{lze}, the zero elements

in I^t_{lze} are replaced by $-(n-1)C$. The replacement of these elements by negative values is going to increase the cost function $g_{lze}(n)$, if the corresponding elements in $I^n_{lze} = S^t_{lze}X^n_{lze}$ are nonnegative. Thus, the number of elements that are negative in both I^{nt}_{lze} and $I^n_{lze} = S^t_{lze}X^n_{lze}$ (are zero in both I^{np}_{lze} and I^t_{lze}) may increase, which may improve the prediction accuracy.

The most important part of each step in the training process is the minimization of $g_{lze}(n)$. The solution X^n_{lze} that minimizes $\|I^{nt}_{lze} - S^t_{lze}X^n_{lze}\|$ is given by [1]:

$$X^n_{lze} = S^{t^+}_{lze}I^{nt}_{lze}, \tag{S2}$$

where $S^{t^+}_{lze}$ is the Moore-Penrose pseudoinverse of S^t_{lze}. Of note, the solution to the minimization problem is not unique and Equation S2 gives the one with minimum norm [1]. This is due to the fact that S^t_{lze} contains redundant information, and consequently its columns are linearly dependent (Text S3). However, if N_{lze} is large enough that the number of linearly independent rows/columns of S^t_{lze} is equal to its maximum possible value, $S_{lze}X^n_{lze}$ is unique for *any* sequence matrix S_{lze} that has been constructed using the same recipe for peptides of the same length (see"Matrix representations and notation"). This is explained in Text S3, which also argues that Equation S2 is still applicable even if S^t_{lze} does not contain maximum possible independent rows/columns.

The multi-step process is terminated when there is no improvement in the prediction accuracy, measured by the median cosine of the angles between the predicted and corresponding experimental intensity vectors. Note that we stop the process when the median cosine (denoted by μ) is maximized, not when $\|I^t_{lze} - I^{np}_{lze}\|$ is minimized. Although these two stopping criteria are related and likely to give similar results, maximizing the median cosine is perhaps better because none of the features used for rescoring (normalized angle, correlation, number of observed and predicted peaks, etc.) depend on the norm of the predicted intensity vectors. To the best of our knowledge, there is no analytical solution to the problem of maximizing median cosine. Hence, in each step the Frobenius norm is minimized, but the steps are stopped when the median cosine is maximized. Instead of cosine, one can use normalized angle or correlation, but these are expected to give similar results. Alternatively, one may generate decoys and terminate the steps when the separation between targets and decoys is maximized, which is the ultimate goal. However, such a process is time consuming. Therefore, we decided to use the simplest measure that is the median cosine. Figure S2 depicts the multi-step training process.

It should be emphasized that minimizing $g_{lze}(n)$ is performed only if the number of peptides with the $l/z/e$ combination is large enough that $I^{nt}_{lze} = S^t_{lze}X^n_{lze}$ is overdetermined. To have an overdetermined system of equations, the number N_{lze} of peptides with $l/z/e$ values, that is the number of rows of S^t_{lze}, must be larger than the number of linearly independent columns/rows of S^t_{lze}. Since there are redundant information in S^t_{lze}, the number of linearly-independent columns (and rows) of S^t_{lze} is smaller than the number M_l of its columns (see Text S2). Thus, if $N_{lze}/M_l \geq 1$, we will have an overdetermined system of equations. However, since there may be many redundant peptides in

the training data, we require $r_{lze} = \tilde{N}_{lze}/M_l \geq 1$, where $\tilde{N}_{lze}$ is the number of *non-redundant* peptides with $l/z/e$ values. Still, if r_{lze} is only slightly larger than 1 overfitting is likely to happen. We address this issue in "Results and discussion". If this criterion is not satisfied (if $r_{lze} < 1$) for $l/z/e$, FastSpel is not trained for this combination.

1.3 Text S3: Number of linearly independent rows of S_{lze}

Consider the sequence matrix S_l representing N_l peptides of length l. (For simplicity, we drop all other indices.) The matrix can be written in block form as $S_l = [s_1, s_2, \ldots, s_l]$, where s_k is an $N_l \times m$ matrix representing the amino acids in the kth position in the peptides' sequences ($m = 21$ is the number of amino acids considered). Denoting the ith column of s_k by s_{ki}, we note that $\sum_i s_{ki} = u$, where u is a vector whose elements are all equal to 1. (Each position in a sequence is occupied by exactly 1 amino acid.) Thus for any $k' \neq k$ and any $j = 1, 2, \ldots, m$ one can write $s_{k'j} = u - \sum_{i \neq j} s_{k'i} = \sum_i s_{ki} - \sum_{i \neq j} s_{k'i}$. In other words, $s_{k'j}$ can be written as a linear combination of the other columns of $s_{k'}$ and the columns of s_k. Thus, columns of S_l are linearly dependent.

1.4 Text S4: General solution to the minimization problem

The general solution to minimizing $\|I^t - S^t X\|$ is given by $X = S^{t^+} I^t + (S^{t^+} S^t - \mathbb{1})w$, where S^{t^+} is the Moore-Penrose pseudoinverse of S^t, $\mathbb{1}$ is the identity matrix, and w is a random matrix of the same size as X [1]. (Again, for simplicity, we have removed all indices except for ^{t}) . The solution would be unique if the columns of S^t were linearly independent, in which case $S^{t^+} S^t = \mathbb{1}$ [1]. But, as mentioned in text S3, the columns of S^t are not linearly independent, and thus X cannot be uniquely determined from minimizing $\|I^t - S^t X\|$. However, if the number of peptides in the training set is large enough that the number of linearly independent rows of S^t is equal to the maximum possible value, for *any* sequence matrix S, the product SX is unique regardless of the choice of X. To see why this is the case we note that X is not unique because, regardless of w, $S^t(S^{t^+} S^t - \mathbb{1})w = 0$, which is in turn due to the fact that $S^t(S^{t^+} S^t - \mathbb{1}) = 0$ [1]. This means that all rows of S^t are perpendicular to all columns of $S^{t^+} S^t - \mathbb{1}$. On the other hand, since S^t contains the maximum possible number of linearly independent rows, any row from any other matrix S that has the same structure as S^t can be written as a linear combination of rows of S^t (these rows span the whole space of vectors representing peptides of length l). Since all rows of S^t are perpendicular to all columns of $S^{t^+} S^t - \mathbb{1}$, all rows of any matrix S with the same structure are also perpendicular to all columns of $S^{t^+} S^t - \mathbb{1}$, and hence $S(S^{t^+} S^t - \mathbb{1}) = 0$. Thus SX, is unique and equal to $SS^{t^+} I^t$ regardless of the value of w. We therefore use the solution $X = S^{t^+} I^t$ that is the one with minimum norm.

Even if the number of peptides in the training set is large, S^t may still have

fewer than maximum number of linearly independent rows. This happens if S^t contains columns with all-zero elements. This is possible because some amino acids may be rarely in certain positions in the sequence. For example, tryptic peptides almost always end with “K” or “R”. If some columns of S^t have no non-zero element, the corresponding rows in X can be anything (including zero) without changing S^tX, because the elements in these rows of X are multiplied by zeros and do not contribute to S^tX at all. In other words, there are no information in the training data about these rows of X, and so they cannot be determined. Now consider another (test) sequence matrix S, for which we want to predict the intensity matrix. We need to choose an X as the solution in which the elements of the undetermined rows all vanish, because they should not contribute to SX. Note that with this choice of X, there will be no contribution from problematic rows of X even if all columns of S have some non-zero values. The solution in which the aforementioned rows of X all vanish is again given by $X = S^{t^+} I^t$. This can be deduced from the fact that $X = S^{t^+} I^t$ is the solution with minimum norm [1], and so must be the solution in which the problematic rows vanish (nonzero values increase the norm). Of note, the fact that some columns of S do not contribute to the intensities may slightly reduce the accuracy of prediction for peptides that include amino acid/position combinations not found in the training data. However, the absence of these amino acid/position combinations in the training data suggests that they rarely happen, and hence few, if any, of the peptides in the test set are expected to contain these amino acid/location combinations. Also, even in peptides that do include these combinations, the intensities can be estimated from the rest of the sequence, and so this issue should have minimal effect on the accuracy of the method. This view is supported by our good results.

1.5 Text S5: Running Percolator

When running Percolator, the “–reset-algorithm” option was specified to run the RESET algorithm, the “-Y” option was applied to make the program use the target-decoy competition method, and the parameter “subset-max-train” was set to 5500000, a value larger than the maximum number of PSMs in the testing datasets (recommended by the Percolator team following an inquiry by the authors). All other parameters were set to the default values.

For Prosit, PeptDeep, and MS2PIP, we used the “rescore.tab” file generated by Oktoberfest, which contains the computed features, as input to Percolator. Since FastSpel currently does not predict retention times, to have a fair comparison between the methods, we also reran Percolator for Prosit, PeptDeep, and MS2PIP excluding the retention time-related features that are the ones computed based on the predicted retention times. The retention time-related features “pred-RT”, “iRT”, “RT”, “abs-rt-diff” constitute only a small fraction of all features calculated by Oktoberfest and used by Percolator. Most features are intensity-related, namely they are calculate using the predicted intensities. There are also general features such as peptide length/charge, collision energy, etc. To distinguish between the Percolator run using all features and the one ex-

cluding the retention time-related features, we denote the former by "IGRTC" (using intensity-related, general, and RT features) and the latter by "IGC" (using intensity-related and general features). The "C" in IGRTC and IGC indicates that the intensities are calculate using the collision energy calibrated methods (not applicable to MS2PIP). Of note, Oktoberfest also uses linear discriminant analysis to compute, using the intensity-related and general features, a new feature ("lda-scores") that is used primarily to align the predicted retention times. Thus, we considered this feature to be retention time-related and excluded it in Percolator (IGC) runs.

To run Percolator for FastSpel, the intensities were first predicted using FastSpel for each PSM. These intensities were then given, as input, to the relevant functions of Oktoberfest to compute the intensity-related features. Finally, the general and computed intensity-related features were fed to Percolator as input. We used Oktoberfest to calculate the features based on FastSpel predictions to have a consistent way of computing the features for the four compared methods.

1.6 Text S6: Summary of symbols

We have introduced many symbols that are used throughout the paper. To make it easier for the reader to find the definitions of the most frequently referenced symbols, they are listed and briefly described below.

- m: Number of amino acids (21)
- l: Peptide length
- z: Peptide charge
- z_f: Fragment charge
- e: Collision energy
- $K_{lz} = 2(l-1)z_{\mathrm{f_{max}}}$: The number of theoretically possible b and y peaks
- S_{lze}: Sequence matrix for the $l/z/e$ combination
- I^t_{lze}: Observed (training) intensity matrix for the $l/z/e$ combination
- I_{lze}: Unfiltered predicted intensity matrix for the $l/z/e$ combination
- X_{lze}: Matrix relating I_{lze} to S_{lze}
- I^p_{lze}: Filtered predicted intensity matrix for the $l/z/e$ combination
- $M_l = lm$: Number of columns of S_{lze}
- N_{lze}: Number of peptides for the $l/z/e$ combination (number of rows of S_{lze})
- $\tilde{N}_{lze}$: Number of non-redundant peptides for the $l/z/e$ combination

- $r_{lze} = \tilde{N}_{lze}/M_l$: Ratio of the number of non-redundant rows of S_{lze} to its number of columns
- α: Normalized angle between the corresponding predicted/observed intensity profiles
- R: Correlation between the corresponding predicted/observed intensity profiles
- n_p: Number of (nonzero) predicted peaks
- n_o: Number of observed peaks
- n_{op}: Number of matched (both observed and predicted) peaks

1.7 Text S7: Prediction speed estimation

To compare the prediction speed of the four methods, we used them to predict the intensity profiles for all PSMs (identified by MaxQuant at the FDR = 100%) in the 22 testing datasets. A fair prediction speed comparison requires running the prediction methods on the same system. On the other hand, Oktoberfest has been developed to run on graphics processing units (GPUs), and FastSpel was designed to be run on central processing units (CPUs). Thus, in practice in may be better to compare the prediction speed of FastSpel run on CPUs with that of Oktoberfest run on GPUs. Hence, we decided to perform both comparisons, namely running FastSpel on CPUs, and running Prosit/PeptDeep/MS2PIP on both CPUs and GPUs.

FastSpel has been developed with on-the-fly calculations in mind. But it could also be used when the input/output are stored in files. In such cases, to have a good estimate of prediction speed, the time spent reading the input and preparing/writing the output should be excluded, especially because FastSpel can predict much faster than it can write. In the case of Oktoberfest, one should consider the fact that Oktoberfest predicts retention times as well. From the output of Oktoberfest, it is not possible to infer the amount of time spent only on intensity prediction. To circumvent this issue, we ran Prosit/PeptDeep/MS2PIP (only intensity prediction) directly on Koina [3], the public server that Oktoberfest uses for its predictions. This was done through "koinapy", the same Python package that Oktoberfest uses. To run the three methods on Koina in the fastest possible way, we set the "mode" parameter to "async" and the "df-output" parameter to "False". With these parameters, Koina, like FastSpel, produces the results in the form of a Python dictionary and no writing or post-processing is involved. The time spent for reading the input was excluded.

To run PeptDeep and MS2PIP on CPUs, we installed their respective Python packages (PeptDeep version 1.3.0; MS2PIP version 4.0.0). Prosit was run through the inSPIRE [2] package version 2.0rc8 (using the "predictSpectra" pipeline). The default parameters were used in all cases. All four prediction methods were run using 1, 8, 16, and 32 CPUs on the Biowulf cluster at the

National Institutes of Health. For each of the 22 testing datasets, a separate job was submitted to the cluster. As a results, for a given dataset, the four methods were run on the same node, but for different datasets they may have been run on various nodes and the times obtained for different datasets may not be comparable. Therefore, for each dataset and each method the relative time, that is the prediction time of the method divided by the prediction time of FastSpel, was computed.

Of note, although FastSpel uses multiprocessing, because of the low computational cost and unavoidable overhead, using multiple CPUs may not noticeably lower the prediction time (or may even increase it). Thus, when FastSpel is used for on-the-fly calculations (no writing), multiprocessing is only used if the number of PSMs is larger than 5 million. Since none of the datasets used in this study contain this many PSMs, multiprocessing was not used for estimating the FastSpel prediction times. For example, when 32 CPUs were used, the relative prediction times of the other three methods were computed by dividing the times spent by the methods divided by the corresponding FastSpel prediction times using only 1 CPU. The relative GPU times were also calculated in the same manner. However, for estimating the total time spent (on reading/prediction/preparing the output/writing) by FastSpel , multiprocessing was used.

For each of the methods the prediction time was estimated as follows. For MS2PIP the time reported by the method was used (excluding reading and writing time). In addition to intensities, PeptDeep predicts retention times and ion mobilities. When run on 1 CPU, PeptDeep reports the times spent on intensity, retention time, and ion mobility prediction separately. We used the reported intensity prediction time in this case. When multiple CPUs are employed, PeptDeep reports only the total prediction time (intensity+retention time+ion mobility). Hence, we used the times obtained with 1 CPU to estimate the percentage of the total time spent on intensity prediction. This percentage was then used to estimate intensity prediction times when multiple CPUs were used. inSPIRE does not report the prediction time at all. Since MS2PIP and inSPIRE use similar input files and produce similar outputs, we assumed the read/write times were comparable for these two methods and to get a rough estimate of the inSPIRE prediction time, we subtracted the MS2PIP read/write time from inSPIRE total time. Given that inSPIRE prediction times are much larger than typical read/write times (see the "Results and discussion" section), the possible errors in these estimates do not change our conclusions.

For completeness, we also compared total times (read+prediction+write) spent by the methods. In the case of PeptDeep, the (estimated) time spent on prediction of retention time and ion mobility was subtracted from the total time. Again, the times were divided by the corresponding FastSpel times to compute the relative times before making comparisons.

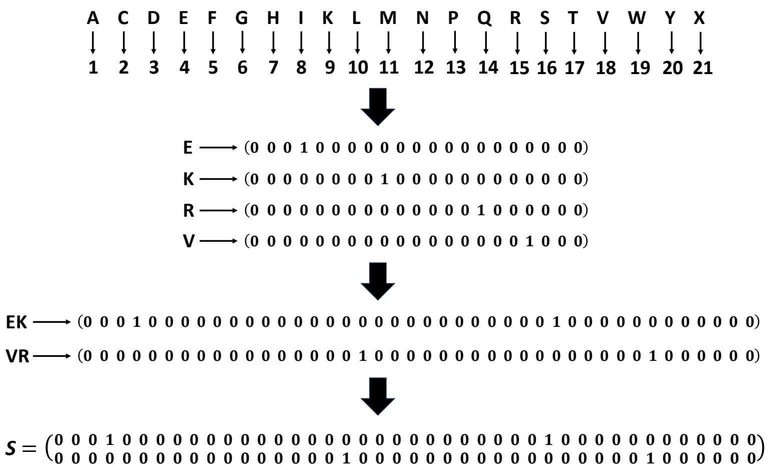

Figure S1: Construction of a sequence matrix. The matrix representation of two peptides EK and VR are shown. For simplicity, the peptides are chosen to have unrealistic lengths of 2. To build this matrix, each amino acid is first assigned an integer and then is represented by a vector based on the integer assigned to it. All elements of this vector vanish except for the one that corresponds to the assigned integer. Examples are shown only for the amino acids present in EK and VR. The vectors corresponding to each amino acid in a peptide are then stacked horizontally to find the peptide vector. The peptide vectors are stacked vertically to construct the sequence matrix S. "X" denotes oxidized methionine.

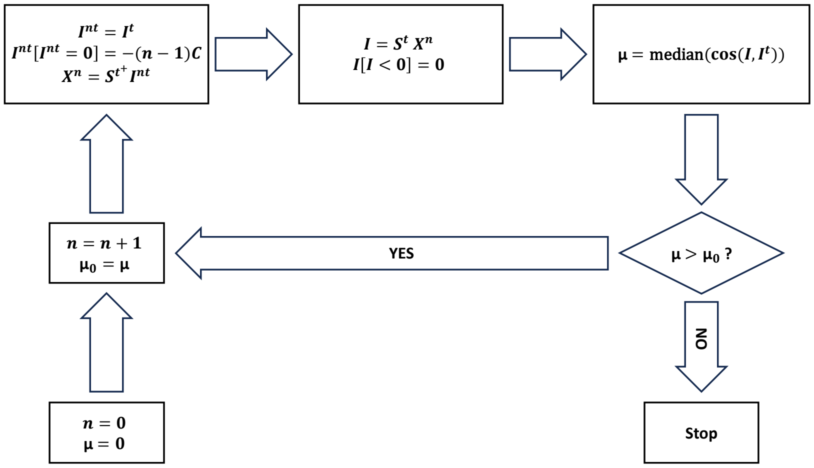

Figure S2: The training workflow. The multi-step training workflow is depicted. Here I^t, S^t, and S^{t^+} respectively denote the observed intensity matrix, the corresponding sequence matrix, and the pseudoinverse of the sequence matrix. $I^{nt}[I^{nt} = 0] = -(n-1)C$ denotes replacing the zero elements of I^{nt} by $-(n-1)C$ (with $C = 0.05$), and $I[I < 0] = 0$ means replacing all negative values in I by zero, where I is the predicted intensity matrix. And $\text{median}(\cos(I, I^t))$ denotes the median of the cosines of the angles between the corresponding rows of the predicted and observed intensity matrices. For simplicity, we have omitted the lze indices.

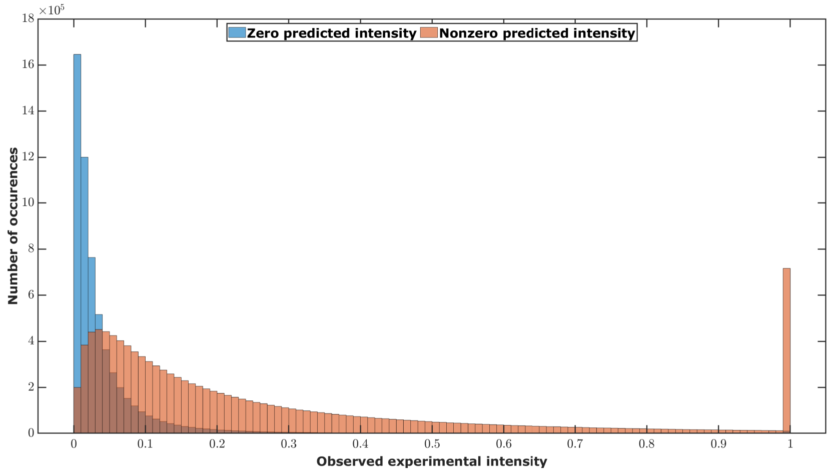

Figure S3: Distributions of the intensities of observed peaks. The distributions are shown for observed peaks with zero and nonzero corresponding predicted intensities. The figure indicates that the observed peaks whose corresponding predicted intensities vanish, are generally small. In fact, 76% of these peaks have intensities less than 0.05. In comparison, 83% of observed peaks that are also predicted have intensities larger than 0.05. Note that all intensity profiles have been normalized to have a maximum of 1. In other words, each profile is guaranteed to contain a value of 1, and hence there is a large maximum at intensity equal to 1.

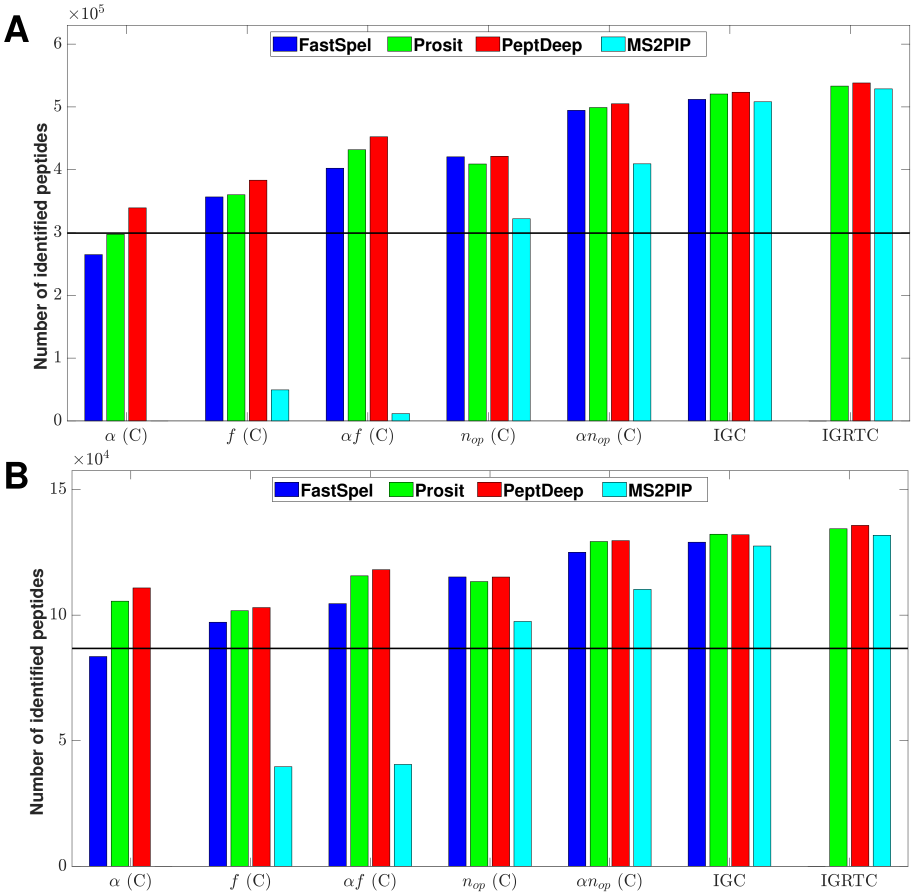

Figure S4: Number of peptides identified by the calibrated prediction methods. The total number of peptides identified using different rescoring methods/functions and the four (collision energy calibrated) prediction approaches are plotted for (A) QE and (B) Fusion instruments. The "(C)" after α, f, αf, n_{op}, and αn_{op} indicates that these scores were calculated using the calibrated prediction methods. Note that IGC, and IGRTC respectively denote Percolator results obtained using intensity-related plus general features, and using all features including retention time-related features. Calibration is not applicable to MS2PIP, and so the values reported here are the same as the ones shown in Figure 5 of the main text. Currently, FastSpel does not have the capability to predict retention times, and so Percolator-IGRTC results are shown for the other three methods. The horizontal line shows the number of peptides identified using the Andromeda score.

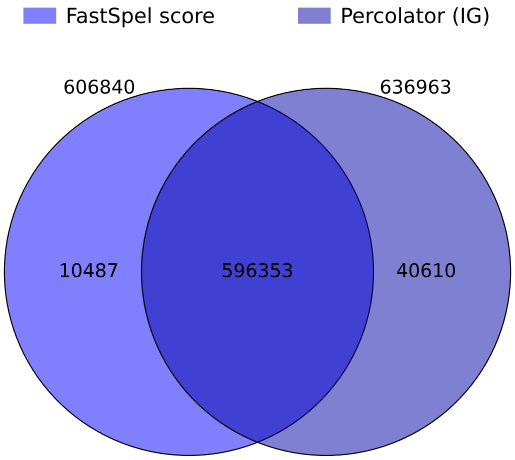

Figure S5: Overlap between the identified peptides. The Venn diagram shows the overlap between peptides identified by FastSpel under two scenarios: (1) rescoring using the new proposed score (αn_{op}). Note that IG indicates that the retention time-related features were excluded and that no collision energy calibration was performed.

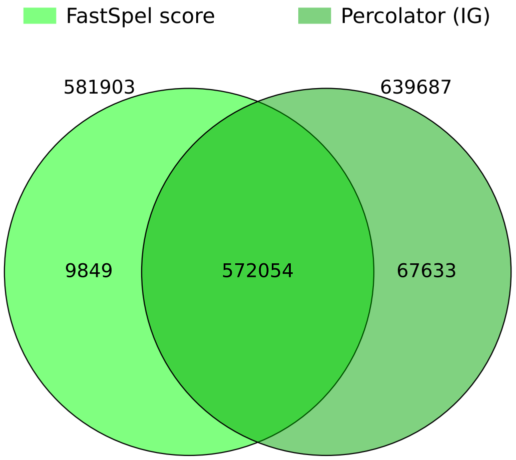

Figure S6: Overlap between the identified peptides. The Venn diagram shows the overlap between peptides identified by Prosit under two scenarios: (1) rescoring using the new proposed score (αn_{op}). Note that IG indicates that the retention time-related features were excluded and that no collision energy calibration was performed.

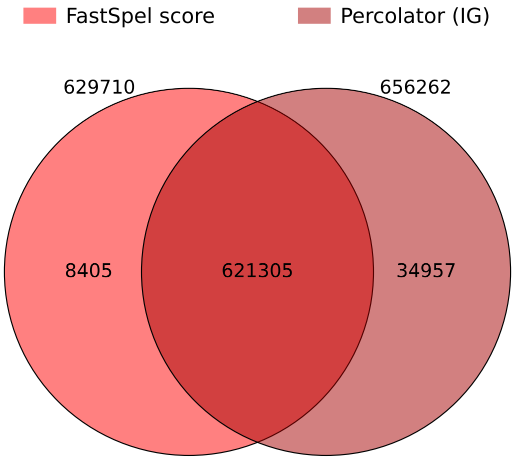

Figure S7: Overlap between the identified peptides. The Venn diagram shows the overlap between peptides identified by PeptDeep under two scenarios: (1) rescoring using the new proposed score (αn_{op}). Note that IG indicates that the retention time-related features were excluded and that no collision energy calibration was performed.

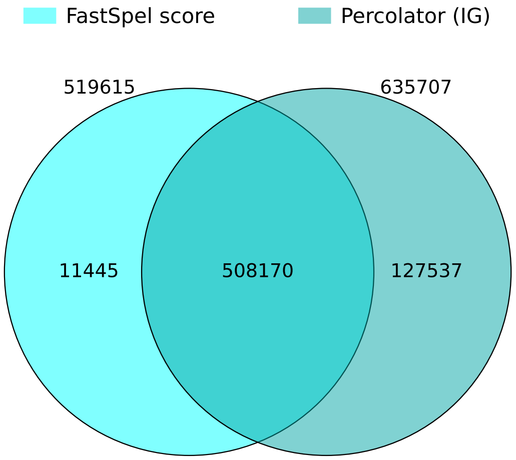

Figure S8: Overlap between the identified peptides. The Venn diagram shows the overlap between peptides identified by MS2PIP under two scenarios: (1) rescoring using the new proposed score (αn_{op}). Note that IG indicates that the retention time-related features were excluded and that no collision energy calibration was performed.

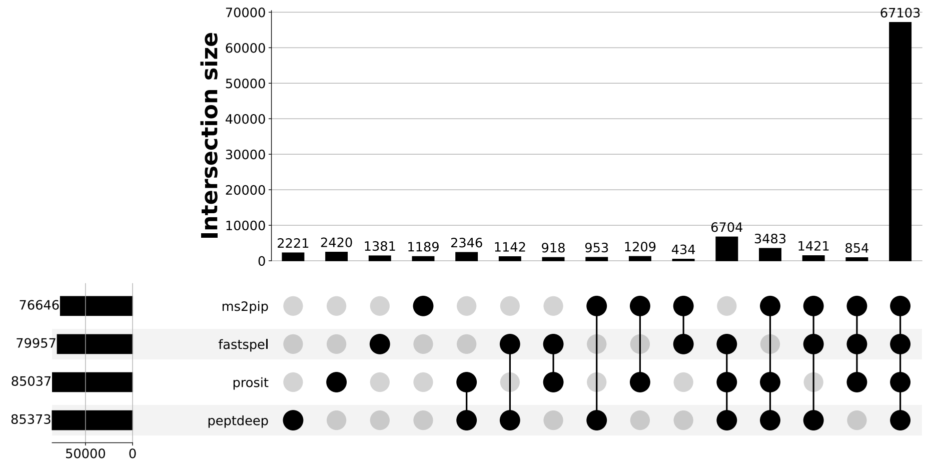

Figure S9: Overlap between peptides identified using DIA-NN. The UpSet plot compares the sets of peptides identified by DIA-NN, when different methods were used to generate the spectral library used for identification. The horizontal bars (lower left) show the total numbers of identified peptides corresponding to each of the four methods (MS2PIP/DIA-NN, FatSpel/DIA-NN, Prosit/DIA-NN, PeptDeep/DIA-NN). The vertical bars show the numbers of peptides exclusively identified by each of the methods or by any combination of them. Peptides exclusively identified by 2 methods, for example, are the ones identified by both of these methods, but missed by the other methods. The (connected) black dot(s) below each vertical bar indicate(s) which method(s) were used to calculate the number of peptides represented by the bar. The last bar (corresponding to the intersection of peptides identified all methods) is very large compared with the others, indicating high overlap between the peptides identified by the four methods.

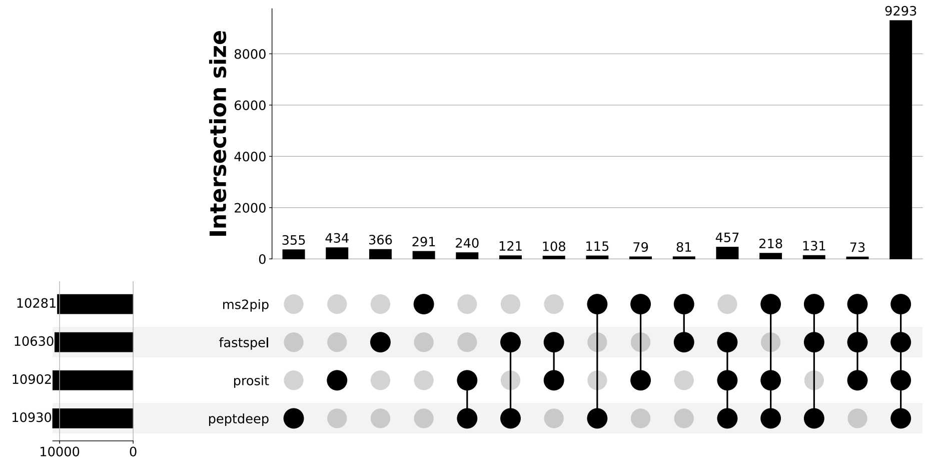

Figure S10: The UpSet plot compares the sets of proteins identified by DIA-NN, when different methods were used to generate the spectral library used for identification. The horizontal bars (lower left) show the total numbers of identified proteins corresponding to each of the four methods (MS2PIP/DIA-NN, FatSpel/DIA-NN, Prosit/DIA-NN, PeptDeep/DIA-NN). The vertical bars show the numbers of proteins exclusively identified by each of the methods or by any combination of them. proteins exclusively identified by 2 methods, for example, are the ones identified by both of these methods, but missed by the other methods. The (connected) black dot(s) below each vertical bar indicate(s) which method(s) were used to calculate the number of proteins represented by the bar. The last bar (corresponding to the intersection of proteins identified all methods) is very large compared with the others, indicating high overlap between the proteins identified by the four methods.

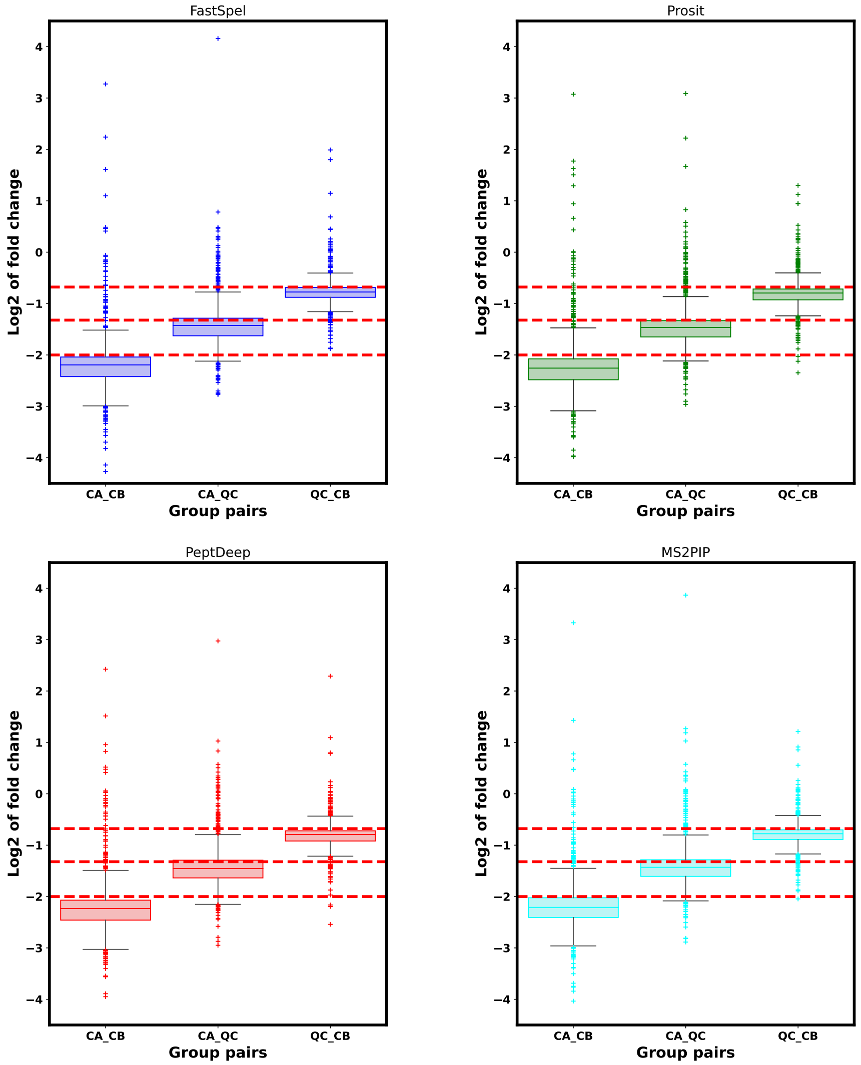

Figure S11: Distributions of log fold changes (Ecoli). For each of the four methods (MS2PIP/DIA-NN, FatSpel/DIA-NN, Prosit/DIA-NN, PeptDeep/DIA-NN), the boxplot shows the distribution of protein fold changes between the three mixtures for Ecoli. The red Horizontal lines depict the expected fold changes based on the experimentally known protein abundance ratios.

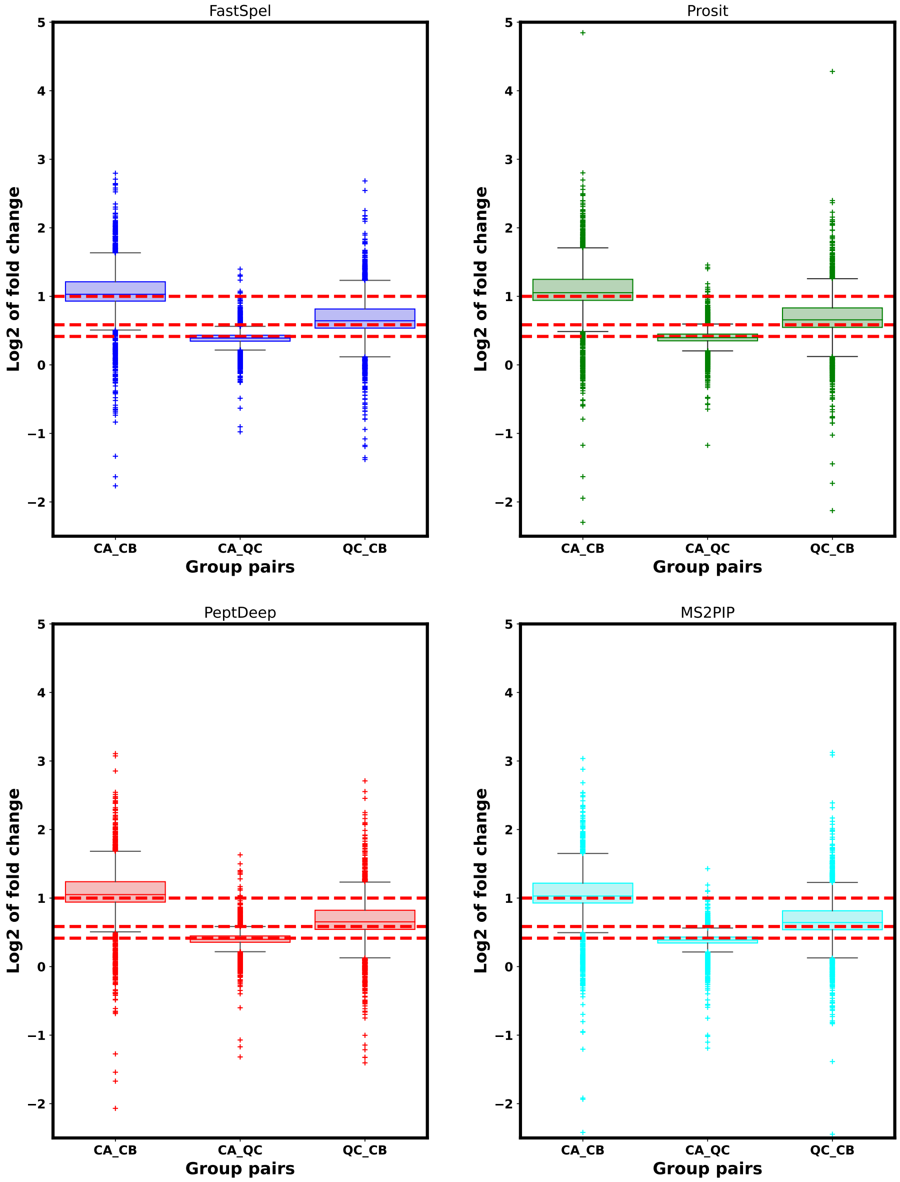

Figure S12: Distributions of log fold changes (Yeast). For each of the four methods (MS2PIP/DIA-NN, FatSpel/DIA-NN, Prosit/DIA-NN, PeptDeep/DIA-NN), the boxplot shows the distribution of protein fold changes between the three mixtures for Yeast. The red Horizontal lines depict the expected fold changes based on the experimentally known protein abundance ratios.

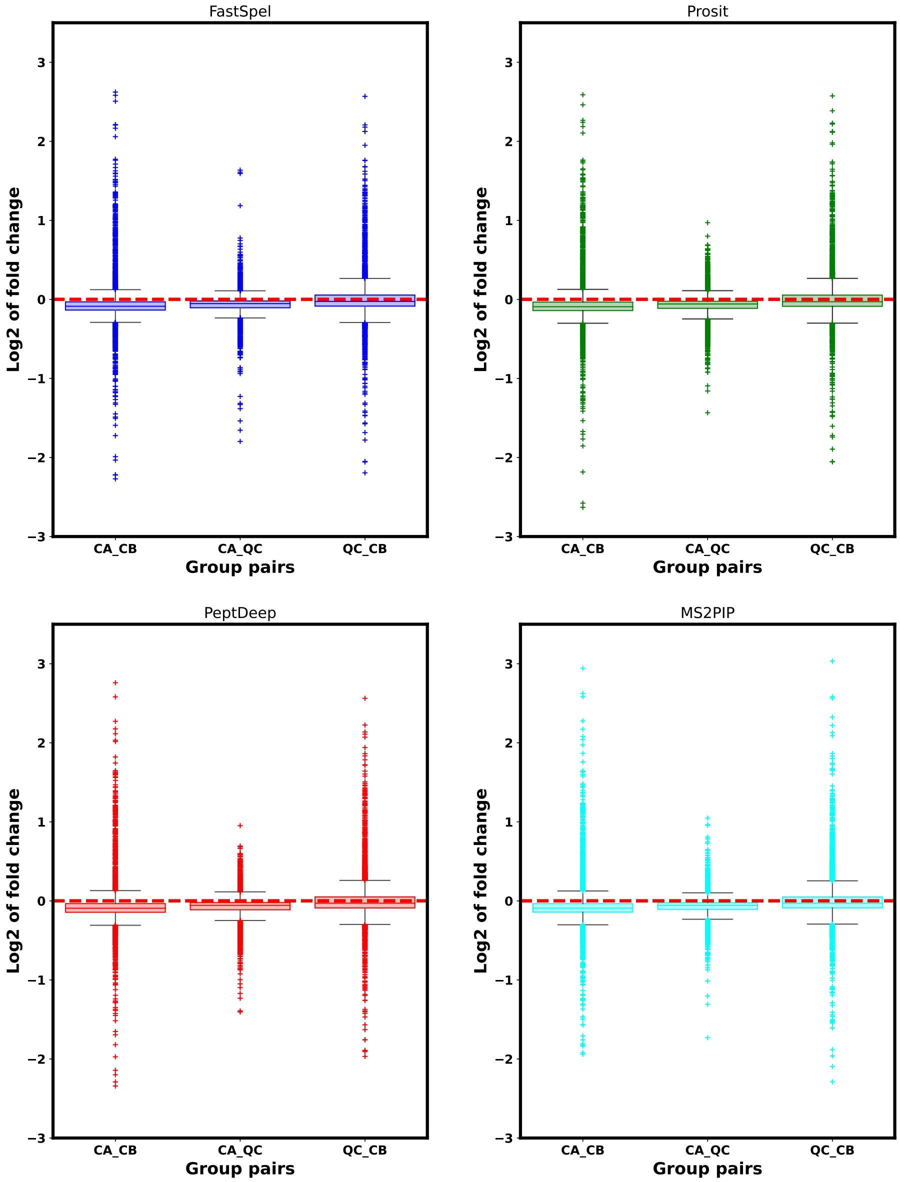

Figure S13: Distributions of log fold changes (Human). For each of the four methods (MS2PIP/DIA-NN, FatSpel/DIA-NN, Prosit/DIA-NN, PeptDeep/DIA-NN), the boxplot shows the distribution of protein fold changes between the three mixtures for Human. The red Horizontal lines depict the expected fold changes based on the experimentally known protein abundance ratios.

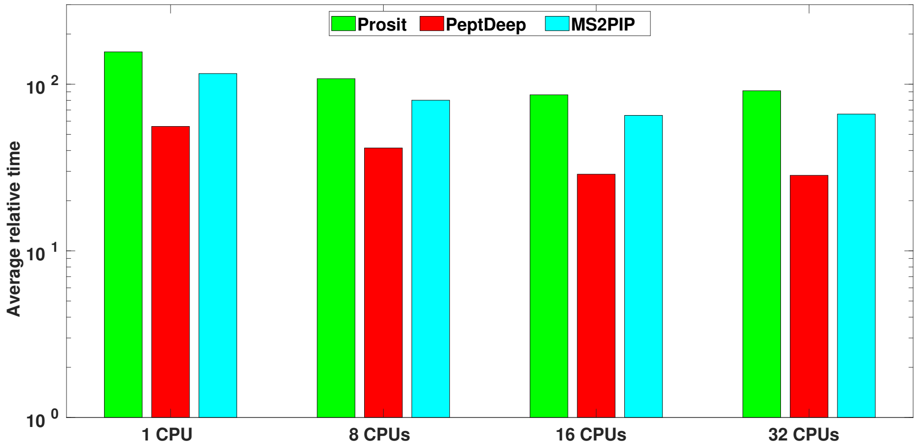

Figure S14: Relative total time. For the three alternative methods the average relative total time spent on spectral library generation (including time spent on reading the input file, making predictions, preparing the output, and writing the output to file) are plotted for various number of CPUs. Note that the times are calculated relative to the time spent by FastSpel, which means FastSpel time in each case is 1 and thus is not shown. The relative total times for 16 and 32 CPUs are almost the same, suggesting that adding more CPUs will not decrease the total relative times for any of the methods.

References

[1] Barata, J. C. A., and Hussein, M. S. The moore–penrose pseudoinverse: A tutorial review of the theory. *Brazilian Journal of Physics 42* (2012), 146–165.

[2] Cormican, J. A., Horokhovskyi, Y., Soh, W. T., Mishto, M., and Liepe, J. inspire: An open-source tool for increased mass spectrometry identification rates using prosit spectral prediction. *Molecular & Cellular Proteomics 21*, 12 (2022).

[3] Lautenbacher, L., Yang, K., Kockmann, T., Panse, C., Chambers, M., Kahl, E., Yu, F., Gabriel, W., Bold, D., Schmidt, T. K., et al. Koina: Democratizing machine learning for proteomics research. *bioRxiv* (2024), 2024–06.
